# Novel Multi-Target Tracking Method: PMBM Filter Combined SVD-SCKF with GP-Driven Measurements

**DOI:** 10.3390/s26092613

**Published:** 2026-04-23

**Authors:** Wentao Jia, Bo Li, Jinyu Zhang, Yubin Zhou

**Affiliations:** 1 School of Electronics and Information Engineering, Liaoning University of Technology, Jinzhou 121001, China; 15666578893@163.com (W.J.); leeboo@yeah.net (B.L.); 2 College of Ocean and Space Information, China University of Petroleum (East China), Qingdao 266000, China; 18753387670@163.com

**Keywords:** Bayesian target discrimination, Gaussian process learning, GP-driven measurement, Poisson multi-Bernoulli mixture filter, random finite set

## Abstract

**Highlights:**

**What are the main findings?**

- Singular value decomposition (SVD)-based square-root cubature Kalman filter (SCKF) prediction with innovation-driven fading factor is embedded in the Poisson multi-Bernoulli mixture (PMBM) recursion. Numerical stability under nonlinear dynamics and target maneuvers is improved, ill-conditioned covariance propagation is avoided, and more reliable state prediction is obtained.
- The measurement mapping and state-dependent uncertainty are learned via a Gaussian process (GP). Then, GP-driven adaptive gating and an in-gate Bayesian measurement-origin test are applied. As a result, clutter-induced misassociations and spurious births are suppressed, multi-target state and cardinality estimates in dense-clutter scenarios are improved.

**What are the implications of the main findings?**

- Numerically stable prediction from square-root nonlinear filtering and GP-based measurement mapping and uncertainty modeling are jointly incorporated within the same PMBM recursion, and robust multi-target tracking results are obtained under measurement-model mismatch and high-clutter conditions.
- A practical pathway for scalable tracking in complex environments is indicated: adaptive gating reduces the effective number of measurements entering association, Bayesian origin discrimination improves association quality, and the applicability to high-clutter, closely spaced, maneuvering-target settings is broadened without prohibitive computational complexity.

**Abstract:**

Owing to multi-target tracking in scenarios with nonlinearity, uncertain measurement model and high clutter density, the Poisson multi-Bernoulli mixture (PMBM) recursion is prone to unstable covariance propagation under nonlinear dynamics as well as uncertainty in measurement-to-target association caused by mismatched gate that causes erroneous updates from clutters. In the prediction stage, the singular value decomposition (SVD) is used in place of Cholesky factorization to construct and propagate the square-root covariance factor in the square-root cubature Kalman filter (SCKF), yielding a numerically stable square-root implementation. Then, the resulting SVD-SCKF is incorporated into the PMBM prediction step and used to propagate the Gaussian-mixture components of both the Poisson point process (PPP) intensity and the Bernoulli component in the Multi-Bernoulli mixture (MBM), yielding predicted means and covariances under nonlinear dynamics. An adaptive fading factor is determined from innovation statistics, and covariance inflation is performed to improve robustness under target maneuvers and model mismatch. In the update stage, the unknown measurement function is regressed by Gaussian process (GP) using historical state–measurement samples, yielding an equivalent measurement mapping and state-dependent uncertainty. Furthermore, the predicted measurement distribution is generated from the GP-based conditional measurement distribution with state prior approximated by SVD-SCKF cubature points. An adaptive gate is determined from the GP-based conditional measurement distribution, which is approximated by an equivalent ellipsoidal gate via fitting for screening the current measurements and filtering out clutter. Residual in-gate clutter measurements are handled via Bayesian target discrimination, where the posterior probability of measurement originated from target is employed as a weight and incorporated into association weights and update likelihoods. Simulation results further confirm the effectiveness and stability of the proposed filter in complex scenarios.

## 1. Introduction

With the development of intelligent transportation, unmanned systems, security surveillance, and smart manufacturing, tracking of multiple moving targets are required for sensors operating in complex environments. Multi-target tracking requires not only estimation of each target’s kinematic state, but also target birth and target death [1,2,3]. In nonlinear scenarios, the measurement-generation process is often non-Gaussian. In practical systems, the measurement function may be unknown, difficult to model accurately, or variable with target states and environmental conditions. Most multi-target tracking methods based on fixed priors are prone to performance degradation [4].

The Bayesian multi-target filter based on random finite set (RFS) [5] has been used to handle variations in the number of targets and the data association between multi-target measurements and tracks, which has become a major solution for multi-target tracking. Within this framework, some filters such as the probability hypothesis density (PHD) filter and cardinalized probability hypothesis density (CPHD) filter [6,7] keep computation tractable by propagating intensity and cardinality statistics. However, the posterior representation is limited because only finite-order statistics are retained. As a result, continuous trajectory output and association consistency are supported by additional methods [8,9]. The multi-Bernoulli (MB) filter [10,11] strengthens target-level posterior representation by modeling target existence with Bernoulli components. The posterior is expressed in a target-component form, and the labeling mechanism is further introduced to form the labeled multi-Bernoulli (LMB) [12] and generalized labeled multi-Bernoulli (GLMB) [13] filters. The different targets are distinguished and trajectory identities are kept from mixing while multiple measurement-to-target association possibilities are represented explicitly during recursion. In an unlabeled point-target modeling setting, the Poisson multi-Bernoulli mixture (PMBM) filter [14] represents undetected targets by Poisson point process and detected targets by multi-Bernoulli mixture. The consistent posterior structure is maintained in recursion and target birth and death, missed detections, and clutter are handled within a unified formulation [15].

The above filters can be implemented in a Gaussian-mixture (GM) form [16] or approximated by sequential Monte Carlo (SMC) [17]. Usually, SMC implementations are affected by particle degeneracy, high cost, and slow tracking response. In contrast, GM parameterization represents the multi-target posterior as a combination of Gaussian components and enables component-wise recursive prediction and update. For nonlinear measurement models, nonlinear Gaussian filters such as the cubature Kalman filter (CKF), unscented Kalman filter (UKF), and the square-root cubature Kalman filter (SCKF) [18,19,20] are embedded to propagate the mean and covariance. The SCKF under measurement-noise uncertainty has also been developed and integrated with the GM-PMBM filter, where covariance propagation is carried out in square-root form to alleviate numerical degradation. However, the existing square-root implementations are built on Cholesky triangular factorizations [21,22] and instability may still arise when the covariance becomes ill-conditioned.

To address the above issues, existing studies have pursued improvements mainly from two directions. On the one hand, factored-form cubature filtering and singular-value-decomposition-based cubature filtering have been developed to enhance the numerical robustness of covariance computation during nonlinear state propagation, thereby alleviating the performance degradation caused by ill-conditioned covariance matrices and unstable matrix factorizations [23,24]. On the other hand, Gaussian process (GP) has been introduced into target tracking for measurement modeling, shape description, and maneuvering-target learning, and have further begun to be combined with PMBM to construct data-driven multi-maneuvering target tracking methods [25,26,27]. However, these advances have still been developed largely along separate technical lines.

Although existing GM-PMBM filters have been strengthened for nonlinear and model-uncertain scenarios, the limitations remain prominent under nonlinearity and dense clutter, that is, the measurement-model mismatch drives the update-stage likelihood away from true distribution and prevents gating from reducing association. To address them, the innovations of this work are threefold:

(1) In the PMBM recursion, each Gaussian component in the PPP and Bernoulli parts is predicted by converting its covariance into a stable square-root using the singular value decomposition (SVD), generating cubature points under SCKF and propagating them through state transition, obtaining the predicted mean and covariance by weighted regression, inflating the predicted covariance using a fading factor under target maneuvers and model mismatch.
(2) In the update stage, the GP is trained using historical state-measurement samples to learn the measurement mapping and provide state-dependent uncertainty, from which a more reliable measurement likelihood is formed for generating update hypotheses and computing their association weights.
(3) The cubature-point approximation of the prior from the prediction stage is combined with the GP-based conditional measurement distributions to yield a predictive measurement distribution. The gating threshold is determined and the resulting region is fitted by an equivalent ellipsoidal gate to remain compatible with PMBM association. The Bayesian measurement test is conducted inside the gate so that the posterior probability that a measurement is target-originated is used as a weight in association and update, reducing the low-credibility measurements on state updating.

The study is organized as follows. Section 2 presents the nonlinear system model and the basic PMBM recursion, and derives the SVD-SCKF prediction with a fading factor and the GP-based measurement model. Section 3 develops and implements the proposed filter, where a complete recursion is provided from numerically stable prediction to GP-based update and clutter association. Section 4 presents the simulation setup and comparative experiments for verifying tracking performance and numerical stability of the proposed filter under challenging conditions. A summary of this study and potential directions for future research are given at the end.

## 2. Preliminaries

### 2.1. System Model

Considering the nonlinear dynamical system, the state and measurement models are

(1) $$\left\{\begin{array}{l} x_{k+1}=f_{k+1|k}\left(x_{k}\right)+u_{k}\\ z_{k+1}=h_{k+1}+v_{k+1} \end{array}\right.$$

where $x_{k+1}$ and $z_{k+1}$ denote the state vector and the measurement vector at time *k* + 1. The nonlinear state-transition function and measurement function are represented by $f_{k+1|k}\left(\cdot \right)$ and $h_{k+1}$, and the process noise $u_{k}$ and measurement noise $v_{k+1}$ are modeled as zero-mean with covariance matrices $Q_{k}$ and $R_{k+1}$. The random finite set (RFS) modeling is employed to represent a time-varying number of targets and the set-valued uncertainty caused by clutter-contaminated measurements.

### 2.2. PMBM Filter

At time *k*, the measurements collected by the sensor are represented as a finite set ${Ζ}_{k}=\left\{z_{1},\ldots ,z_{n}\right\}$, and the multi-target state is represented as a finite set $Y_{k}=\left\{x_{1},\cdots ,x_{l}\right\}$. The measurement set ${Ζ}_{k}$ is modeled as a union of target-originated detections and clutter returns, where clutter is characterized by an intensity function $\kappa \left(z\right)$.

Under the RFS formulation, the PMBM filter represents the multi-target posterior using two complementary RFS components: a Poisson RFS for the set of undetected targets $Y^{u}$ and a multi-Bernoulli mixture (MBM) RFS for the set of detected targets $Y^{d}$. The multi-target state Y is expressed as the disjoint union of two parts, leading to the standard PMBM factorization:

(2) $$f\left(Y\right)=\sum_{Y^{u}⊎Y^{d}=Y}f^{p}\left(Y^{u}\right)f^{mbm}\left(Y^{d}\right)$$

The Poisson RFS density $f^{p}$ with intensity $\mu \left(\cdot \right)$ and the MBM RFS density $f^{mbm}$ are defined as

(3) $$f^{p}\left(Y\right)=e^{-\int \mu \left(x\right)dx}\prod_{x\in Y}\mu \left(x\right)$$

(4) $$f^{mbm}\left(Y\right)\propto \sum_{j\in I}\sum_{⊎Y^{l}=Y}\prod_{l=1}^{n}w^{j,l}f^{j,l}\left(Y^{l}\right)$$

(5) $$f^{j,l}\left(Y^{l}\right)=\left\{\begin{array}{l} r^{j,l}p^{j,l}\left(x\right),\;\;Y=\left\{x\right\}\\ 1-r^{j,l},\;\;Y=\emptyset \;\\ 0,\;\;\;\;\mathrm{else}\; \end{array}\right.$$

The MBM RFS density is interpreted as a normalized weighted sum of multi-Bernoulli multi-target densities and is parameterized by ${\left\{w^{j,l},{\left\{r^{j,l},p^{j,l}\left(x\right)\right\}}_{l\in I^{j}}\right\}}_{j\in I}$ for each global hypothesis j∈I, where $I^{j}$ denotes the index set of Bernoulli components in the *j*-th multi-Bernoulli. Given the measurement set ${Ζ}_{k}$, the PMBM recursion is carried out through both the prediction step and update step.

### 2.3. SVD Implementation of SCKF

In the PMBM recursion framework, both the PPP and Bernoulli components are parameterized using GM. Consequently, under a nonlinear state transition, it is essential to propagate the means and covariances of the Gaussian components in a numerically stable manner. To this end, an SVD-based implementation of SCKF is necessary.

#### 2.3.1. Prediction

At time *k*, given the nonlinear system state estimate ${\bar{x}}_{k}$ and covariance matrix $P_{k}$, an SVD factorization is performed as

(6) $$P_{k}=U_{k}\Sigma_{k}U_{k}^{T}$$

where $\Sigma_{k}$ is the singular-value (diagonal) matrix of $P_{k}$, $U_{k}$ the left singular vector matrix of $P_{k}$, and ${\left(\cdot \right)}^{T}$ denotes matrix transpose.

Firstly, the cubature points are generated by

(7) $$X_{i,k}=U_{k}\Sigma_{k}^{1/2}\xi_{i}+{\bar{x}}_{k}$$

where ${\bar{x}}_{k}$ is the state estimate at time *k*, $\xi_{i}=\sqrt{n}{\left[1\right]}_{i}$ is the *i*-th cubature point, n is the state dimension, and $\left[1\right]$ is defined as

(8) $$\left[1\right]=\left[\begin{array}{llllll} \left(\begin{array}{c} 1\\ 0\\ \vdots \\ 0 \end{array}\right) & \ldots & \left(\begin{array}{c} 0\\ 0\\ \vdots \\ 1 \end{array}\right) & \left(\begin{array}{c} -1\\ 0\\ \vdots \\ 0 \end{array}\right) & \ldots & \left(\begin{array}{c} 0\\ 0\\ \vdots \\ -1 \end{array}\right) \end{array}\right]$$

where the *i*-th cubature point corresponds to the *i*-th column of the point-set matrix. The cubature points are propagated through the nonlinear state-transition function as

(9) $$X_{i,k+1|k}^{*}=f_{k+1|k}\left(X_{i,k}\right)$$

and the predicted state mean is obtained by the weighted fusion of the propagated cubature points:

(10) $${\bar{x}}_{k+1|k}=\frac{1}{2n}\sum_{i=1}^{2n}X_{i,k+1|k}^{*}$$

Note that the covariance square-root is constructed via SVD instead of Cholesky to improve numerical stability under ill-conditioned cases. Appendix A provides the error bound for perturbation propagation to justify the robustness of this replacement.

#### 2.3.2. Update

At time *k*, the prior covariance of the nonlinear system is

(11) $$\begin{array}{ll} P_{k+1|k}^{*} & =E\left[\left(f_{k+1|k}\left(X_{i,k}\right)+u_{k}-{\bar{x}}_{k+1|k}\right){\left(f_{k+1|k}\left(X_{i,|k}\right)+u_{k}-{\bar{x}}_{k+1|k}\right)}^{T}\right]\\ & =E\left[\left(X_{i,k+1|k}^{*}-{\bar{x}}_{k+1|k}\right){\left(X_{i,k+1|k}^{*}-{\bar{x}}_{k+1|k}\right)}^{T}+u_{k}u_{k}^{T}\right] \end{array}$$

The SVD factorization is then applied to $P_{k+1|k}^{*}$ as

(12) $$P_{k+1|k}^{*}=U_{k+1|k}\Sigma_{k+1|k}V_{k+1|k}^{T}$$

and the cubature points after nonlinear state mapping are expressed as

(13) $$X_{i,k+1|k}=U_{k+1|k}\Sigma_{k+1|k}^{1/2}\xi_{i,k}+{\bar{x}}_{k+1|k}$$

The cubature points in the measurement space are obtained through the nonlinear measurement function:

(14) $$Z_{i,k+1|k}=h_{k+1}\left(X_{i,k+1|k}\right)$$

and the predicted measurement is computed by averaging these points:

(15) $${\bar{z}}_{k+1|k}=\frac{1}{2n}\sum_{i=1}^{2n}Z_{i,k+1|k}$$

The fading factor $\lambda_{k+1}$ is introduced to artificially inflate the predicted error covariance $P_{k+1|k}^{*}$ at the linearization point. Greater weight is assigned to the current observations, and less reliance is placed on historical information, which supports faster tracking of state variations.

The covariance of propagated measurements is defined as

(16) $$\begin{array}{ll} P_{zz,k+1|k}^{*} & =E\left[\left(Z_{i,k+1}-{\bar{z}}_{k+1|k}\right){\left(Z_{i,k+1}-{\bar{z}}_{k+1|k}\right)}^{T}\right]\\ & =E\left[\left(\lambda_{k+1}^{1/2}H_{k+1}\left(X_{i,k+1}-{\bar{x}}_{k+1|k}\right)+v_{k+1}\right){\left(\lambda_{k+1}^{1/2}H_{k+1}\left(X_{i,k+1}-{\bar{x}}_{k+1|k}\right)+v_{k+1}\right)}^{T}\right]\\ & =\lambda_{k+1}H_{k+1}E\left[\left(X_{i,k+1}^{*}-{\bar{x}}_{k+1|k}\right){\left(X_{i,k+1}^{*}-{\bar{x}}_{k+1|k}\right)}^{T}+u_{k}u_{k}^{T}\right]H_{k+1}^{T}+E\left[v_{k+1}v_{k+1}^{T}\right]\\ & =\lambda_{k+1}H_{k+1}P_{k+1|k}^{*}H_{k+1}^{T}+R_{k+1} \end{array}$$

where $H_{k+1}$ is the Jacobian of the nonlinear measurement function with respect to $X_{i,k}$:

(17) $$\frac{\partial H_{\mathrm{GP},k+1}}{\partial X_{i,k}}=H_{k+1}$$

Note that $H_{\mathrm{GP},k+1}$ denotes the function used for calculating the Jacobian $H_{k+1}$ measurement mapping, which is learned from an alternative model based on GP. At the initial stage, due to insufficient training data, the initial measurement model $H_{\mathrm{GP},0}$ is used for initialization. Once the training set reaches the minimum required size $L_{\min}$, $H_{\mathrm{GP},k+1}$ obtained from GP is used to replace $H_{\mathrm{GP},0}$ and its measurement uncertainty that varies with state. At the same time, $R_{k+1}$ should be replaced with $R_{\mathrm{GP},k+1}$. For simplicity, $R_{k+1}$ is used in all equations in this work, and $R_{0}$ is used for initialization.

The fading factor $\lambda_{k+1}$ is computed from (16):

(18) $$\begin{array}{cc} \lambda_{k+1}H_{k+1}P_{k+1|k}^{*}H_{k+1}^{T} & =E\left[\left(Z_{i,k+1}-{\bar{z}}_{k+1|k}\right){\left(Z_{i,k+1}-{\bar{z}}_{k+1|k}\right)}^{T}\right]-R_{k+1}\\ \lambda_{k+1}\mathrm{tr}\left(H_{k+1}P_{k+1|k}^{*}H_{k+1}^{T}\right) & =\mathrm{tr}\left(E\left[\left(Z_{i,k+1}-{\bar{z}}_{k+1|k}\right){\left(Z_{i,k+1}-{\bar{z}}_{k+1|k}\right)}^{T}\right]-R_{k+1}\right)\\ \lambda_{k+1} & =\frac{\mathrm{tr}\left(E\left[\left(Z_{i,k+1}-{\bar{z}}_{k+1|k}\right){\left(Z_{i,k+1}-{\bar{z}}_{k+1|k}\right)}^{T}\right]-R_{k+1}\right)}{\mathrm{tr}\left(H_{k+1}P_{k+1|k}^{*}H_{k+1}^{T}\right)} \end{array}$$

To improve numerical robustness by avoiding negative values and to restrict the adjustment to uncertainty inflation only, (18) is modified as

(19) $$\lambda_{k+1}=\max\left\{1,\frac{\mathrm{tr}\left(E\left[\left(Z_{i,k+1}-{\bar{z}}_{k+1|k}\right){\left(Z_{i,k+1}-{\bar{z}}_{k+1|k}\right)}^{T}\right]-R_{k+1}\right)}{\mathrm{tr}\left(H_{k+1}P_{k+1|k}^{*}H_{k+1}^{T}\right)}\right\}$$

Then (16) is revised accordingly:

(20) $$P_{zz,k+1|k}=\lambda_{k+1}H_{k+1}P_{k+1|k}^{*}H_{k+1}^{T}+\left(1-\lambda_{k+1}\right)Q_{k}H_{k+1}^{T}+R_{k+1}$$

Similarly, the cross-covariance between the propagated state and measurement is defined:

(21) $$\begin{array}{ll} P_{xz,k+1|k}^{*} & =E\left[\lambda_{k+1}^{1/2}\left(X_{i,k+1}-{\bar{x}}_{k+1|k}\right){\left(Z_{i,k+1}-{\bar{z}}_{k+1|k}\right)}^{T}\right]\\ & =E\left[\lambda_{k+1}^{1/2}\left(X_{i,k+1}-{\bar{x}}_{k+1|k}\right){\left(\lambda_{k+1}^{1/2}H_{k+1}\left(X_{i,k+1}-{\bar{x}}_{k+1|k}\right)+v_{k+1}\right)}^{T}\right]\\ & =E\left[\lambda_{k+1}^{1/2}\left(f_{k+1|k}\left(X_{i,k}\right)+u_{k}-{\bar{x}}_{k+1|k}\right){\left(\lambda_{k+1}^{1/2}\left(f_{k+1|k}\left(X_{i,k}\right)+u_{k}-{\bar{x}}_{k+1|k}\right)+v_{k+1}\right)}^{T}\right]H_{k+1}^{T}\\ & =\lambda_{k+1}E\left[\left(X_{i,k+1}^{*}-{\bar{x}}_{k+1|k}\right){\left(X_{i,k+1}^{*}-{\bar{x}}_{k+1|k}\right)}^{T}+u_{k}u_{k}^{T}\right]H_{k+1}^{T}\\ & =\lambda_{k+1}P_{k+1|k}^{*}H_{k+1}^{T} \end{array}$$

To keep the noise covariance in the filtering process consistent with $Q_{k}$, it is corrected as

(22) $$P_{xz,k+1|k}=\lambda_{k+1}P_{k+1|k}^{*}H_{k+1}^{T}+\left(1-\lambda_{k+1}\right)Q_{k}H_{k+1}^{T}$$

The Kalman gain is then given by

(23) $$K_{k+1}=P_{xz,k+1|k}/P_{zz,k+1|k}$$

and the state estimate is updated as

(24) $${\bar{x}}_{k+1}=K_{k+1}\left(Z_{k+1}-{\bar{z}}_{k+1|k}\right)+{\bar{x}}_{k+1|k}$$

The predicted covariance is rewritten in expectation form as

(25) $$\begin{array}{ll} P_{k+1|k} & =E\left[\lambda_{k+1}^{1/2}\left(X_{i,k+1}-{\bar{x}}_{k+1|k}\right)\lambda_{k+1}^{1/2}{\left(X_{i,k+1}-{\bar{x}}_{k+1|k}\right)}^{T}\right]\\ & =\lambda_{k+1}E\left[\left(f_{k+1|k}\left(X_{i,k}\right)+u_{k}-{\bar{x}}_{k+1|k}\right){\left(f_{k+1|k}\left(X_{i,|k}\right)+u_{k}-{\bar{x}}_{k+1|k}\right)}^{T}\right]\\ & =\lambda_{k+1}E\left[\left(X_{i,k+1|k}^{*}-{\bar{x}}_{k+1|k}\right){\left(X_{i,k+1|k}^{*}-{\bar{x}}_{k+1|k}\right)}^{T}+u_{k}u_{k}^{T}\right]\\ & =\lambda_{k+1}P_{k+1|k}^{*} \end{array}$$

When model mismatch occurs and $f_{k+1|k}\left(\cdot \right)$ and $h_{k+1}\left(\cdot \right)$ become inaccurate, the innovation $\left(Z_{i,k+1}-{\bar{z}}_{k+1|k}\right)$ increases and yields $\lambda_{k+1}>1$, which enlarges $K_{k+1}$ and assigns higher confidence to the current observation $Z_{i,k+1}$, leading to faster correction of state estimate and reduced estimation error. When an abrupt state change occurs and the state exhibits a sudden jump, the predicted mean ${\bar{x}}_{k+1|k}$ may deviate markedly from the true value and cause an abnormal increase in $\left(Z_{i,k+1}-{\bar{z}}_{k+1|k}\right)$, producing a large $\lambda_{k+1}$ and rapidly amplifying $K_{k+1}$ so that the jump can be tracked. Then, the updated covariance is written in expectation form as

(26) $$\begin{array}{ll} P_{k+1}^{*} & =E\left[\lambda_{k+1}^{1/2}\left(X_{i,k+1}-{\bar{x}}_{k+1}\right)\lambda_{k+1}^{1/2}{\left(X_{i,k+1}-{\bar{x}}_{k+1}\right)}^{T}\right]\\ & =E\left[\lambda_{k+1}^{1/2}\left(\left(X_{i,k+1}-{\bar{x}}_{k+1|k}\right)-K_{k+1}\left(Z_{i,k+1}-H_{k+1}{\bar{x}}_{k+1|k}\right)\right)\lambda_{k+1}^{1/2}{\left(\left(X_{i,k+1}-{\bar{x}}_{k+1|k}\right)-K_{k+1}\left(Z_{i,k+1}-H_{k+1}{\bar{x}}_{k+1|k}\right)\right)}^{T}\right]\\ & =E\left[\left(\lambda_{k+1}^{1/2}\left(I-K_{k+1}H_{k+1}\right)\left(X_{i,k+1}-{\bar{x}}_{k+1|k}\right)-K_{k+1}v_{k+1}\right){\left(\lambda_{k+1}^{1/2}\left(I-K_{k+1}H_{k+1}\right)\left(X_{i,k+1}-{\bar{x}}_{k+1|k}\right)-K_{k+1}v_{k+1}\right)}^{T}\right]\\ & =\lambda_{k+1}\left(I-K_{k+1}H_{k+1}\right)P_{k+1|k}^{*}{\left(I-K_{k+1}H_{k+1}\right)}^{T}+K_{k+1}R_{k+1}K_{k+1}^{T}\\ & =\lambda_{k+1}\left(P_{k+1|k}^{*}-P_{k+1|k}^{*}H_{k+1}^{T}K_{k+1}^{T}-K_{k+1}H_{k+1}P_{k+1|k}^{*}+P_{k+1|k}^{*}H_{k+1}^{T}K_{k+1}^{T}\right)\\ & =\lambda_{k+1}\left(I-K_{k+1}H_{k+1}\right)P_{k+1|k}^{*} \end{array}$$

The similar correction is applied, yielding

(27) $$P_{k+1}=\left(I-K_{k+1}H_{k+1}\right)\lambda_{k+1}P_{k+1|k}^{*}+\left(I-\left(I-K_{k+1}H_{k+1}\right)\lambda_{k+1}\right)Q_{k}$$

**Remark 1.**  *Although the SVD is a useful algorithm, the SVD-based SCKF is incorporated into the PMBM recursion. It is organically combined with the adaptive fading factor to improve robustness under maneuvering and model mismatch that is novel in the current literature.*

### 2.4. GP Learning of Measurement Function

For the known state-transition model, stable state prediction and covariance propagation can be achieved by the SVD-SCKF. In practical applications, the measurement function is unknown or highly nonlinear and the statistical properties of measurement noise vary with target state. Then, GP is introduced to learn the measurement function and the measurement-noise covariance in a data-driven manner, providing informative measurement priors for the GP–SVD–PMBM framework.

When the true measurement function $h_{k+1}$ is unknown, GP regression is adopted for modeling, and the latent function is characterized from limited training data through similarity relationships among samples. Any finite-dimensional marginal distribution induced by a GP is Gaussian and latent function values at test inputs are inferred from training samples, enabling data-driven learning of both measurement function and measurement-noise covariance. Each GP training datum is assumed to describe an input–output relation. The standard measurement model is given by $z=h\left(x\right)+v,v\sim N\left(0,\sigma_{v}^{2}\right)$, and the training set is $D=\left\{X,Z\right\}={\left\{\left(X_{i},Z_{i}\right)\right\}}_{i=1}^{2n}$, where *X* denotes the true-state matrix and *Z* denotes the measurement–output matrix.

**Proposition 1.**  *Suppose that* $h\left(x\right)∼GP\left(H_{\mathrm{GP},k+1},R_{\mathrm{GP},k+1}\right)$ *at time k, GP-based approximations of* h *and* $R_{k+1}$ *are expressed as*

(28) $$\left\{\begin{array}{l} h≐H_{\mathrm{GP},k+1}=K_{i,k+1|k}^{T}{\left[K\left(X_{k+1|k},X_{k+1|k}\right)+\sigma_{v}^{2}I\right]}^{-1}Z_{k+1|k}\\ R_{k+1}≐R_{\mathrm{GP},k+1}=K_{**}-K_{*}^{T}{\left(K\left(X_{k+1|k},X_{k+1|k}\right)+\sigma_{v}^{2}I\right)}^{-1}K_{*} \end{array}\right.$$

**Proof.**  For the arbitrary RFS $X=\left\{X_{1},X_{2},\cdots ,X_{2n}\right\}$, the noise-free measurement-value vector $h={\left[h\left(X_{1}\right),h\left(X_{2}\right),\cdots ,h\left(X_{2n}\right)\right]}^{T}$ is modeled as a multivariate Gaussian:

(29) $$p\left(h|X\right)=N\left(0,K\right)$$

Each entry $K_{ij}$ in the kernel matrix K is evaluated at the inputs $X_{i}$ and $X_{j}$ through the Gaussian kernel function $K_{ij}=K\left(X_{i},X_{j}\right)$, where $K\left(\cdot ,\cdot \right)$ is the squared-exponential kernel:

(30) $$K_{ij}=K\left(X_{i},X_{j}\right)=\sigma_{X}^{2}\exp\left(-\frac{{\left\|X_{i}-X_{j}\right\|}^{2}}{2l^{2}}\right)$$

where $\sigma_{X}^{2}$ is the signal variance that scales the covariance magnitude, ${\left\|X_{i}-X_{j}\right\|}^{2}$ is the squared Euclidean distance between $X_{i}$ and $X_{j}$. The small distances yield covariances close to the maximum and indicate strong correlation, while large distances drive the covariance toward zero and indicate near independence. The length-scale parameter is a positive *d*-dimensional vector $L={\left[L_{1},L_{2},\cdots ,L_{d}\right]}^{T}$ that determines the characteristic scale of each input dimension. The same length-scale to all dimensions yields an isotropic squared-exponential covariance function.

The conditional distribution of the measurement is modeled as

(31) $$p\left(z|h\right)=N\left(h,\sigma_{v}^{2}I\right)$$

and the joint distribution implied by (29) and (30) is written as

(32) $$p\left(z,h|X\right)=p\left(z|h\right)p\left(h|X\right)$$

Marginalization over (33) gives

(33) $$p\left(z|X\right)=\int p\left(z|h\right)p\left(h|X\right)dh$$

With measurement noise v and the closure of Gaussian distributions under convolution, the measurement is distributed as

(34) $$z=h+v\sim N\left(0,K+\sigma_{v}^{2}I\right)$$

The log marginal likelihood is then expressed as

(35) $$\log p\left(z|X\right)=-\frac{1}{2}z^{T}{\left(K+\sigma_{v}^{2}I\right)}^{-1}z-\frac{1}{2}\log\left|K+\sigma_{v}^{2}I\right|-n\log2\pi$$

During GP training, the latent value $H\left(x_{*}\right)$ at a test input $x_{*}$ is predicted from the training set, and the joint prior is modeled as a Gaussian distribution:

(36) $$\left[\begin{array}{c} h\\ h_{*} \end{array}\right]\sim N\left(0,\left[\begin{array}{cc} K & K_{*}\\ K_{*}^{T} & K_{**} \end{array}\right]\right)$$

where $K_{*}={\left[K\left(x_{*},x_{1}\right),K\left(x_{*},x_{2}\right),\cdots ,K\left(x_{*},x_{2n}\right)\right]}^{T}$ and $K_{**}=K\left(x_{*},x_{*}\right)$.

With measurement noise included, the joint distribution becomes

(37) $$\left[\begin{array}{c} z\\ h_{*} \end{array}\right]\sim N\left(\left[\begin{array}{c} 0\\ 0 \end{array}\right],\left[\begin{array}{cc} K+\sigma_{v}^{2}I & K_{*}\\ K_{*}^{T} & K_{**} \end{array}\right]\right)$$

The conditional predictive distribution is Gaussian:

(38) $$p\left(z|h\right)=N\left(0+K_{*}^{T}{\left(K+\sigma_{v}^{2}I\right)}^{-1}\left(z-0\right),K_{**}-K_{*}^{T}{\left(K+\sigma_{v}^{2}I\right)}^{-1}K_{*}\right)$$

The kernel width and GP hyperparameters are collected in $\theta =\left\{\sigma_{x},\sigma_{v},l\right\}$ and are estimated by maximizing the log marginal likelihood:

(39) $$\theta_{\max}=\underset{\theta }{\arg\max\log}\;p\left(z|X,\theta \right)$$

The required gradients are obtained from (35) and are evaluated using a numerical optimization scheme:

(40) $$\frac{\partial \log p\left(z|X,\theta \right)}{\partial \theta_{i}}=-\frac{1}{2}z^{T}{\left(K+\sigma_{v}^{2}I\right)}^{-1}\frac{\partial \left(K+\sigma_{v}^{2}I\right)}{\partial \theta_{i}}{\left(K+\sigma_{v}^{2}I\right)}^{-1}z-\frac{1}{2}\mathrm{tr}\left({\left(K+\sigma_{v}^{2}I\right)}^{-1}\frac{\partial \left(K+\sigma_{v}^{2}I\right)}{\partial \theta_{i}}\right)$$

(41) $$\frac{\partial \log p\left(z|X,\theta \right)}{\partial l}=-\frac{1}{2}z^{T}{\left(K+\sigma_{v}^{2}I\right)}^{-1}\frac{\partial \left(K+\sigma_{v}^{2}I\right)}{\partial \theta_{i}}{\left(K+\sigma_{v}^{2}I\right)}^{-1}z-\frac{1}{2}\mathrm{tr}\left({\left(K+\sigma_{v}^{2}I\right)}^{-1}\frac{\partial \left(K+\sigma_{v}^{2}I\right)}{\partial l}\right)$$

(42) $$\frac{\partial \log p\left(z|X,\theta \right)}{\partial \sigma_{X}}=-\frac{1}{2}z^{T}{\left(K+\sigma_{v}^{2}I\right)}^{-1}\frac{\partial \left(K+\sigma_{v}^{2}I\right)}{\partial \sigma_{X}}{\left(K+\sigma_{v}^{2}I\right)}^{-1}z-\frac{1}{2}\mathrm{tr}\left({\left(K+\sigma_{v}^{2}I\right)}^{-1}\frac{\partial \left(K+\sigma_{v}^{2}I\right)}{\partial \sigma_{X}}\right)$$

(43) $$\frac{\partial \log p\left(z|X,\theta \right)}{\partial \sigma_{v}}=-\frac{1}{2}z^{T}{\left(K+\sigma_{v}^{2}I\right)}^{-1}\frac{\partial \left(K+\sigma_{v}^{2}I\right)}{\partial \sigma_{v}}{\left(K+\sigma_{v}^{2}I\right)}^{-1}z-\frac{1}{2}\mathrm{tr}\left({\left(K+\sigma_{v}^{2}I\right)}^{-1}\frac{\partial \left(K+\sigma_{v}^{2}I\right)}{\partial \sigma_{v}}\right)$$

where tr(·) denotes the matrix trace, defined as the sum of diagonal elements. At time *k*, $X_{k+1|k}$, $Z_{k+1|k}$, and $X_{i,k+1|k}$ in the PMBM recursion are substituted into X, Z, and $x_{*}$, and the GP-based model can provide explicit approximations. □

## 3. Proposed Filter

### 3.1. Overall Architecture

**Figure 1:**
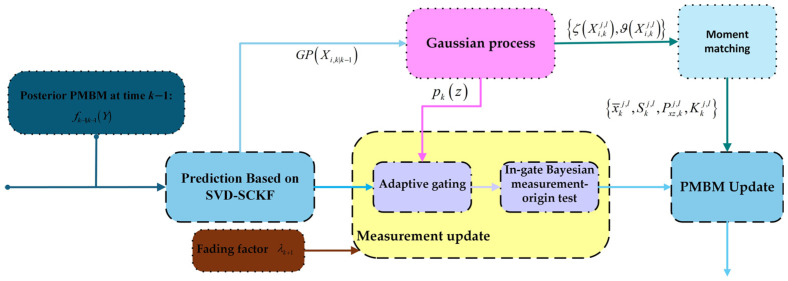
Prediction and update of proposed filter.

Numerical instability under strong maneuvers and measurement-model mismatch in cluttered environments is addressed by proposing GP–SVD–PMBM–SCKF multi-target filter, and overall workflow is illustrated in Figure 1.

In the prediction stage, the SVD-SCKF is incorporated. The SVD is performed on the covariance square root to improve numerical stability and preserve directional characteristics when ill-conditioned matrices are encountered. The fading factor is introduced to adaptively adjust the predicted covariance so that higher weight is assigned to current observations and less dependence is placed on historical estimates when rapid state variations occur or the model becomes inaccurate.

In the update stage, the GP learning mechanism is incorporated, where a training set is constructed from predicted states and their associated historical measurements. The similarity between inputs is quantified by a kernel function, and GP-based predictions of the mean and covariance of measurement function output are obtained. It provides a more accurate characterization of the measurement distribution and its uncertainty, and improving consistency between update-stage measurement estimation and the actual sensing process.

The GP-derived measurement prior is also used to construct a data-driven measurement gating region. In a conventional PMBM filter, a chi-square ellipsoidal gate is typically formed from the predicted measurement mean and covariance, and the resulting gate is fully determined by both linearized model and assumed noise statistics, which can be insufficient for describing the true contour of measurement clusters under complex non-Gaussian conditions. With the GP predictive distribution, the more accurate probability density in the measurement space is obtained and an adaptive ellipsoidal gate is formed through iso-density contours.

### 3.2. Prediction Based on SVD-SCKF

RFS integrals arising in the PMBM filter are intractable in nonlinear systems, and a Gaussian-filter implementation is adopted in which the Bernoulli state density is approximated by a Gaussian mixture, converting the set-integral operations in the RFS domain into recursive computations over a finite number of Gaussian components.

Under the Gaussian distribution $N\left(\cdot \right)$, the GM form of Poisson intensity for newborn targets is

(44) $$\beta_{k}^{b}\left(x\right)=\sum_{m=1}^{J_{k}^{\beta }}w_{k}^{\beta ,m}N\left(x;{\bar{x}}_{k}^{\beta ,m},P_{k}^{\beta ,m}\right)$$

where $J_{k}^{b}$ is the number of Gaussian components, $w_{k}^{b,m}$ is the weight of the *m*-th component, ${\bar{x}}_{k}^{b,m}$ and $P_{k}^{b,m}$ are the mean and covariance, respectively.

At time *k* − 1, the PPP intensity is represented in GM form as

(45) $$\mu_{k-1}\left(x\right)=\sum_{m=1}^{J_{k-1}^{\mu }}\omega_{k-1}^{\mu ,m}N\left(x;{\bar{x}}_{k-1}^{\mu ,m},P_{k-1}^{\mu ,m}\right)$$

And the predicted PPP intensity at time *k* is expressed as

(46) $$\mu_{k|k-1}\left(x\right)=\beta_{k}^{b}\left(x\right)+\sum_{m=1}^{J_{k-1}^{\mu }}w_{k|k-1}^{\mu ,m}N\left(x;{\bar{x}}_{k|k-1}^{\mu ,m},P_{k|k-1}^{\mu ,m}\right)$$

With the predicted weight defined by

(47) $$w_{k|k-1}^{\mu ,m}=w_{k-1}^{\mu ,m}p_{S,k}$$

According to (47), ${\bar{x}}_{k|k-1}^{\mu ,m}$ and $P_{k|k-1}^{\mu ,m}$ are defined as

(48) $$X_{m,k-1}={\bar{x}}_{k}^{\mu ,m}+\sqrt{P_{k-1}^{j,m}}\alpha_{m}$$

(49) $${\bar{x}}_{k|k-1}^{\mu ,m}=\frac{1}{2n}\sum_{i=1}^{2n}X_{i,k+1|k}$$

(50) $${\bar{x}}_{k|k-1}^{j,l}=\frac{1}{2n}\sum_{i=1}^{2n}X_{i,k+1|k}^{j,l}$$

(51) $$P_{k|k-1}^{\mu ,m}=\frac{1}{2n}\sum_{i=1}^{2n}\left(X_{i,k+1|k}-{\bar{x}}_{k|k-1}^{\mu ,m}\right){\left(X_{i,k+1|k}-{\bar{x}}_{k|k-1}^{\mu ,m}\right)}^{T}+Q_{k}$$

For the *l*-th Bernoulli component at time *k* − 1, the state density is

(52) $$p_{k-1}^{j,l}\left(x\right)=N\left(x;{\bar{x}}_{k-1}^{j,l},P_{k-1}^{j,l}\right)$$

The predicted existence probability is updated as

(53) $$r_{k|k-1}^{j,l}=r_{k-1}^{j,l}p_{S,k}$$

And the component weight is kept unchanged:

(54) $$w_{k|k-1}^{j,l}=w_{k-1}^{j,l}$$

The predicted mean ${\bar{x}}_{k|k-1}^{j,l}$ and covariance $P_{k|k-1}^{j,l}$ are computed from the propagated cubature points as

(55) $${\bar{x}}_{k|k-1}^{j,l}=\frac{1}{2n}\sum_{i=1}^{2n}X_{i,k+1|k}^{j,l}$$

(56) $$P_{k|k-1}^{j,l}=\frac{1}{2n}\sum_{i=1}^{2n}\left(X_{i,k+1|k}^{j,l}-{\bar{x}}_{k|k-1}^{j,l}\right){\left(X_{i,k+1|k}^{j,l}-{\bar{x}}_{k|k-1}^{j,l}\right)}^{T}+Q_{k}^{j,l}$$

(57) $$X_{k+1|k}^{j,l}={\bar{x}}_{k}^{j,l}+\sqrt{P_{k|k+1}^{j,l}}\alpha_{m}$$

The cubature points are constructed using SVD and propagated with the prescribed weights, yielding stable nonlinear prediction while preserving the GM-PMBM filter. Algorithm A1 (see Appendix B) summarizes the prediction process of the SVD-SCKF adopted in this section.

### 3.3. Measurement Gating and Classification

The traditional PMBM uses a fixed ellipsoidal gate to filter measurements. The gate is built in the measurement space using the mean and covariance of the predicted measurement. For each candidate measurement, the Mahalanobis distance to the predicted measurement is calculated. The measurement is kept only if this distance is smaller than a preset threshold. In that case, it is considered statistically consistent with the target prediction and is used for later data association. Otherwise, it is discarded. When the measurement model is nonlinear or suffers from model mismatch, the true target-generated measurement distribution may deviate from this fixed ellipsoidal approximation. This may lead to inaccurate gating results. To address this issue, a GP-based predictive measurement distribution is introduced. The predicted mean and covariance obtained from GP are used to construct an adaptive ellipsoidal gate. This gate is used to reject measurements that are unlikely to be target-originated. For the measurements inside the gate, the posterior probability of target origin is computed from the predictive measurement distribution and the clutter model. This probability is further used to modulate the association likelihood, so that low-confidence clutter measurements have less influence on the PMBM update.

#### 3.3.1. GP-Driven Gating Region Construction

The measurement distribution conditioned on the known state $x_{k}$ is

(58) $$p\left(z_{k}|x_{k},D\right)=N\left(\varsigma_{GP}\left(x_{k}\right),\vartheta_{GP}\left(x_{k}\right)\right)$$

where $\varsigma_{GP}\left(x_{k}\right)$ and $\vartheta_{GP}\left(x_{k}\right)$ denote the GP posterior predictive conditional mean and conditional covariance:

(59) $$\;\varsigma_{GP}(x_{k})=K_{*}^{T}{\left(K+\sigma_{v}^{2}I\right)}^{-1}\left(z-0\right)$$

(60) $$\vartheta_{GP}(x_{k})=K_{**}-K_{*}^{T}{\left(K+\sigma_{v}^{2}I\right)}^{-1}K_{*}$$

Note that Equations (59) and (60) characterize measurement distribution at a single-target state. The prediction errors are typically present in the target state. A single fixed point is not assumed. The target state is represented by a prior density. The conditional measurement density is combined with the state prior. The predicted measurement density is obtained, and the gate construction is performed from this predicted density:

(61) $$p_{k}\left(z\right)≜p\left(z_{k}|Z_{1:k-1}\right)=\int p\left(z_{k}|x_{k}\right)p\left(x_{k}|Z_{1:k-1}\right)dx_{k}$$

where $p\left(z_{k}|Z_{1:k-1}\right)$ denotes the prior state density, and $p\left(z_{k}|x_{k}\right)$ denotes the conditional measurement density. In SVD-SCKF, the prior state density is approximated by a finite set of cubature points ${\left\{X_{i,k},w_{k}^{i}\right\}}_{i=1}^{2n}$:

(62) $$p\left(x_{k}|Z_{1:k-1}\right)\approx \sum_{i=1}^{2n}w_{k}^{i}\delta \left(x_{k}-X_{i,k}\right)$$

For each cubature point $X_{i,k}$, the corresponding conditional measurement distribution is provided by the GP posterior:

(63) $$p\left(z_{k}|x_{k}=X_{i,k},D\right)=N\left(z_{k};\varsigma_{GP}\left(X_{i,k}\right),\vartheta_{GP}\left(X_{i,k}\right)\right)$$

Substituting Equation (63) into Equation (61), it gives an approximate form of the predicted measurement density:

(64) $$\begin{array}{ll} p_{k}\left(z\right) & =\int p\left(z_{k}|x_{k},D\right)p\left(x_{k}|Z_{1:k-1}\right)dx_{k}\approx \int p\left(z_{k}|x_{k},D\right)\sum_{i=1}^{2n}w_{k}^{i}\delta \left(x_{k}-X_{i,k}\right)dx_{k}\\ & =\sum_{i=1}^{2n}w_{k}^{i}p\left(z_{k}\left|x_{k}=X_{i,k}\right.\right) \end{array}$$

The predicted measurement density is represented as a Gaussian mixture with weighted local Gaussian components associated with the cubature points:

(65) $$p_{k}\left(z\right)\approx \sum_{i=1}^{2n}w_{k}^{i}N\left(z_{k};\varsigma_{GP}\left(X_{i,k}\right),\vartheta_{GP}\left(X_{i,k}\right)\right)$$

The predicted measurement mean is defined as

(66) $${\bar{z}}_{k}^{GP}=\sum_{i=1}^{2n}w_{k}^{i}\varsigma_{GP}\left(X_{i,k}\right)$$

The predicted measurement covariance is given by

(67) $$S_{k}^{GP}=\sum_{i=1}^{2n}w_{k}^{i}\left[\vartheta_{GP}\left(X_{i,k}\right)+\left(\varsigma_{GP}\left(X_{i,k}\right)-{\bar{z}}_{k}^{GP}\right){\left(\varsigma_{GP}\left(X_{i,k}\right)-{\bar{z}}_{k}^{GP}\right)}^{T}\right]$$

The ellipsoidal gating based on the Mahalanobis distance is retained in the PMBM association stage. An adaptive ellipsoidal gate is constructed using the predicted measurement mean and covariance:

(68) $$G_{k}^{GP}=\left\{z\in R^{d_{z}}:{\left(z-{\bar{z}}_{k}^{GP}\right)}^{T}S_{k}^{GP-1}{\left(\varsigma_{GP}\left(X_{l,k}\right)-{\bar{z}}_{k}^{GP}\right)}^{T}\leq \iota \right\}$$

where ${\bar{z}}_{k}^{GP}$ and $S_{k}^{GP-1}$ determine the gate center and scale. ι is a threshold selected by the gating probability. The PMBM association structure is kept unchanged. The gate center and scale are adjusted by the state-dependent measurement distribution.

#### 3.3.2. Probabilistic Clutter Assessment Inside the Gate

Measurement gating removes observations that are unlikely to be generated by the target, while measurements that pass the gate may still be clutter. Using the predictive measurement distribution $p_{k}(z)$, the reliability of in-gate measurements is assessed, and for any $z\in G_{k}^{ellip}$, the normalized predictive density is defined as

(69) $$\pi_{k}(z)=\frac{p_{k}(z)}{\underset{z^{\prime }\in R^{d_{z}}}{\max}p_{k}(z^{\prime })}$$

Note that a larger $\pi_{k}(z)$ indicates that the measurement lies in a high-density region of the predictive distribution and is therefore more likely to be a true target-originated measurement. When $\pi_{k}(z)$ is small yet the measurement still falls within the ellipsoidal gate, it satisfies the geometric distance constraint but matches the predictive distribution poorly. Consequently, it is statistically unlikely to be generated by the target model and may correspond to in-gate clutter. The larger $\pi_{k}(z)$ indicates that the measurement lies in a high-density region of the predictive distribution and is more likely to be target-originated. While a small $\pi_{k}(z)$ with *z* still inside the ellipsoidal gate indicates that the geometric distance constraint is satisfied but the match to the predictive distribution is weak. The measurement is statistically unlikely to be generated by the target model and may correspond to in-gate clutter. The normalized density $\pi_{k}(z)$ provides a probabilistic measure of consistency between an in-gate measurement and the predicted target distribution. However, the explicit threshold is still required to separate high-likelihood target measurements from clutter located in low-density regions. Such a threshold is chosen empirically, making it difficult to maintain consistency across different scenarios. To avoid the uncertainty introduced by an empirical threshold, the reliability assessment of in-gate measurements is reformulated as a binary Bayesian hypothesis-testing problem [28]. The normalized density $\pi_{k}(z)$ quantifies consistency between an in-gate measurement and the target predictive distribution, and a threshold is still required to separate high-likelihood target measurements from clutter in low-density regions. To avoid dependence on an empirically selected threshold, the reliability assessment is formulated as a binary Bayesian hypothesis-testing problem.

Suppose that $\eta_{T}$ denotes the hypothesis that the measurement is target-originated and $\eta_{C}$ denotes the hypothesis that the measurement is clutter for any in-gate measurement:

(70) $$p(z|\eta_{T},Z_{1:k-1})=p_{k}(z)$$

(71) $$p(z|\eta_{C})=\kappa \left(z\right)$$

where $p_{k}(z)$ is the predictive measurement distribution.

Given the prior probabilities $p(\eta_{T})=\pi_{T}$ and $p(\eta_{C})=\pi_{C}$ to ensure consistency between the hypothesis test and the PMBM generative model, the prior for the target hypothesis is set as $\pi_{T}\propto r_{k}P_{D}P_{g}$, and the prior for the clutter hypothesis is set as where $\pi_{C}\propto \lambda_{c}V_{g}$, $\lambda_{c}$ is the clutter rate and $V_{g}$ is the gating volume. Note that $p_{k}(z)$ denotes the predictive measurement distribution. Prior probabilities are set as $p(\eta_{T})=\pi_{T}$ and $p(\eta_{C})=\pi_{C}$ to keep consistency with the hypothesis test and the PMBM generative model, where $\pi_{T}\propto r_{k}P_{D}P_{g}$ is used for the target-originated hypothesis and $\pi_{C}\propto \lambda_{c}V_{g}$ is used for the clutter hypothesis, with $\lambda_{c}$ being the clutter rate and $V_{g}$ the gating volume. The posterior probability that an in-gate measurement is target-originated is then written as

(72) $$p\left(\eta_{T}|z,Z_{1:k-1}\right)=\frac{\pi_{T}p_{k}\left(z\right)}{\pi_{T}p_{k}\left(z\right)+\pi_{C}\kappa \left(z\right)}$$

The association likelihood is modulated by this reliability weight:

(73) $$g_{k,i}^{eff}\left(z_{k}\right)=p\left(\eta_{T}|z,Z_{1:k-1}\right)g_{k,i}\left(z_{k}\right)$$

Using $P\left(\eta_{T}|z,Z_{1:k-1}\right)$ as the probabilistic reliability weight down-weights measurements in low-density regions during the update and assigns larger association weights to high-confidence measurements, while the PMBM association structure is kept unchanged and the influence of in-gate clutter is reduced in complex scenarios.

### 3.4. GP-Based Update

For a measurement $z_{k}$ at the current time that is not associated with any existing Bernoulli component, the PMBM filter uses the measurement-predicted intensity $e_{k}\left(z_{k}\right)$ of newborn targets to represent the predicted likelihood that $z_{k}$ is explained by the Poisson birth intensity, yielding the existence probability $r_{k}^{p}$ of a newborn target. Conditioned on the hypothesis that this measurement corresponds to a newborn target, the posterior state density $p^{p}\left(x_{k}|z_{k}\right)$ is obtained by updating the PPP Gaussian-mixture components. In the computation of $e_{k}\left(z_{k}\right)$, the Bayesian discriminant probability of the measurement-generation hypothesis is incorporated as a weighting factor, thereby reducing the probability that clutter measurements are initialized as newborn targets.

Algorithm A2 (see Appendix C) summarizes the GP-aided cubature update procedure used in this section. Each cubature point is fed into the GP posterior predictor to obtain $\zeta \left(\cdot \right)$ and $\vartheta \left(\cdot \right)$, which are moment-matched to form $\bar{x}$, S, $P_{xz}$, and for the PMBM update in (74)–(84).

(74) $$r_{k}^{p}=e_{k}\left(z_{k}\right)/\rho^{p}\left(z_{k}\right)$$

(75) $$p^{p}\left(x_{k}|z_{k}\right)=\frac{1}{e_{k}\left(z_{k}\right)}\sum_{m=1}^{J_{k}^{\mu }}w_{k|k-1}^{\mu ,m}N\left(x;{\bar{x}}_{k}^{p,m},P_{k}^{p,m}\right)$$

The associated parameters are defined as

(76) $$\left\{\begin{array}{l} e_{k}\left(z_{k}\right)=\sum_{m=1}^{J_{k|k-1}^{\mu }}w_{k}^{m}p\left(\eta_{T}|z,Z_{1:k-1}\right)N\left(z;{\bar{x}}_{k}^{m},P_{k}^{m}\right)\\ \rho^{p}\left(z_{k}\right)=\kappa \left(z\right)+e_{k}\left(z_{k}\right)\\ {\bar{x}}_{k}^{p,m}={\bar{x}}_{k}^{\mu ,m}+K_{k}^{p}\left(z_{k}-{\bar{x}}_{k}^{m}\right)\\ P_{k}^{p,m}=P_{k}^{\mu ,m}-K_{k}^{p}S_{k}^{p}{\left(K_{k}^{p}\right)}^{T}\\ X_{i,k}^{p}={\bar{x}}_{k}^{\mu ,m}+\sqrt{P_{k|k+1}^{\mu ,m}}\alpha_{i}\\ {\bar{x}}_{k}^{m}=\frac{1}{2n}\sum_{i=1}^{2n}z_{k|k-1}^{p,i} \end{array}\right.$$

(77) $$\left\{\begin{array}{l} S_{k}^{p}=\frac{1}{2n}\sum_{i=1}^{2n}\left(X_{i,k}^{p}-{\bar{x}}_{k}^{m}\right)\cdot {\left(X_{i,k}^{p}-{\bar{x}}_{k}^{m}\right)}^{T}+R_{k}^{p}\\ P_{k}^{m}=S_{k}^{p}\\ P_{xz,k}^{p}=\frac{1}{2n}\sum_{i=1}^{2n}\left({\bar{x}}_{k|k-1}^{\mu ,m}-X_{i,k}^{p,m}\right)-{\left({\bar{x}}_{k}^{m}-z_{k|k-1}^{p,i}\right)}^{T}\\ K_{k}^{p}=P_{xz,k}^{p}{\left(S_{k}^{p}\right)}^{-1} \end{array}\right.$$

In the update stage, uncertainties caused by missed detections and false alarms are accounted for through explicit modeling. The Bayesian-consistent updates of the target existence probability and the state density are obtained:

(78) $$w_{k}^{j,l,mis}=w_{k|k-1}^{j,l}\left(1-r_{k|k-1}^{j,l}+r_{k|k-1}^{j,l}\left(1-p_{D}\left(x\right)\right)\right)$$

(79) $$r_{k}^{j,l,mis}=\frac{r_{k|k-1}^{j,l}\left(1-p_{D}\left(x\right)\right)}{1-r_{k|k-1}^{j,l}+r_{k|k-1}^{j,l}\left(1-p_{D}\left(x\right)\right)}$$

(80) $$p_{k}^{j,l,mis}\left(x\right)=N\left(x;{\bar{x}}_{k}^{j,l,mis},P_{k}^{j,l,mis}\right)$$

where ${\bar{x}}_{k}^{j,l,mis}={\bar{x}}_{k|k-1}^{j,l}$ and $P_{k}^{j,l,mis}=P_{k|k-1}^{j,l}$.

For a measurement $z_{k}$ associated with an existing Bernoulli component under the data-association hypothesis, the potentially detected target is updated using the measurement–track association branch weight $w_{k}^{j,l}\left(z\right)$. It is determined by the track prior, the existence probability, and the predicted measurement likelihood. The Bayesian discriminant probability for the measurement-generation hypothesis is included as a multiplicative factor to modulate the predicted measurement likelihood magnitude. The measurements that pass the gate but are statistically more consistent with clutter are down-weighted through this discriminant term:

(81) $$w_{k}^{j,l}\left(z\right)=w_{k|k-1}^{j,l}P\left(\eta_{T}|z,Z_{1:k-1}\right)r_{k|k-1}^{j,l}N\left(z;{\bar{x}}_{k}^{j,l,u},P_{k}^{j,l,u}\right)$$

(82) $$r_{k}^{j,l}\left(z\right)=1$$

(83) $$p_{k}^{j,l}\left(x|z\right)=N\left(x;{\bar{x}}_{k}^{j,l},P_{k}^{j,l}\right)$$

The associated parameters are defined as follows:

(84) $$\left\{\begin{array}{l} {\bar{x}}_{k}^{j,l}={\bar{x}}_{k|k-1}^{j,l}+K_{k}^{u}\left(z_{k}-{\bar{x}}_{k}^{j,l}\right)\\ K_{k}^{u}=P_{xz,k}^{u}{\left(S_{k}^{u}\right)}^{-1}\\ P_{xz,k}^{u}=\frac{1}{2n}\sum_{i=1}^{2n}\left({\bar{x}}_{k|k-1}^{j,l}-X_{i,k}^{u}\right)-{\left({\bar{x}}_{k}^{j,l}-z_{k|k-1}^{u,i}\right)}^{T}\\ S_{k}^{u}=\frac{1}{2n}\sum_{i=1}^{2n}\left(X_{i,k}^{u}-{\bar{x}}_{k}^{j,l}\right){\left(X_{i,k}^{u}-{\bar{x}}_{k}^{j,l}\right)}^{T}+R_{k}^{u}\\ {\bar{x}}_{k}^{j,l,u}=\frac{1}{2n}\sum_{i=1}^{2n}z_{k|k-1}^{u,i}\\ P_{k}^{j,l,u}=S_{k}^{u}\\ X_{i,k}^{u}={\bar{x}}_{k}^{j,l}+\sqrt{P_{k|k-1}^{j,l}}\alpha_{i} \end{array}\right.$$

### 3.5. Algorithm Summary and Complexity

Computational complexity of the proposed GP–SVD–PMBM filter is analyzed by examining GP learning, GP-based measurement prediction, the resulting measurement gating and in-gate probability evaluation, and the data-association cost in the update stage.

#### 3.5.1. Computational Cost of GP-Based Measurement Processing

In proposed framework, the GP is used mainly for measurement prediction in the update stage. GP training-set size is denoted by *L*. State dimension is $n_{x}$. In SVD-SCKF, $2n_{x}$ cubature points are selected for each component to be updated. GP posterior prediction is evaluated once at each cubature point. Conditional measurement mean and conditional measurement covariance are obtained. For a single test point, the kernel vector between the test point and all *L* training samples is computed. Predictive mean and predictive covariance are solved. With kernel-matrix factorization available, computational complexity of GP posterior prediction at the single test point is commonly denoted as $O\left(L^{2}\right)$. At time *k*, the number of PPP Gaussian components and the number of Bernoulli components participating in the update are denoted by $J_{k}$ and $N_{k}$. The total number of components requiring measurement prediction is $J_{k}+N_{k}$. Each component is associated with $2n_{x}$ cubature points. Total number of GP posterior predictions in the one-step update is approximated by $2n_{x}\left(J_{k}+N_{k}\right)$. Total additional complexity introduced by GP in the one-step update is

(85) $$C_{\mathrm{GP},\mathrm{up}}=O\left(2n_{x}\left(J_{k}+N_{k}\right)L^{2}\right)$$

After predicted measurement mean and covariance are obtained, measurements are screened by equivalent ellipsoidal gating. One Mahalanobis distance test is required for each track–measurement pair. Suppose the measurement dimension is denoted by $d_{z}$, the per-test complexity is $O\left(d_{z}\right)$. For two-dimensional measurements, this complexity is regarded as constant order. The total gating complexity is $O\left(N_{k}M_{k}{d_{z}}^{2}\right)$. In the two-dimensional case, approximation $O\left(N_{k}M_{k}\right)$ is used.

For each gated-in measurement z, probability density $p\left(z\right)$ under the predicted measurement distribution is evaluated. Statistical consistency with the target prediction is quantified. The density $p\left(z\right)$ is represented as the weighted sum of $O\left(2n_{x}\right)$. Suppose total number of gated-in track–measurement pairs is $G_{k}$, the total number of gated-in track–measurement pairs is $O\left(2n_{x}G_{k}\right)$. Suppose the number of gated-in measurements for track ο is $n_{k}^{ο}$, the total complexity is written as $C_{\mathrm{in}\text{-}\mathrm{gate}}=O\left(N_{k}\cdot 2n_{x}\right)$.

The complexity of GP learning and gate-related computation is determined mainly by state dimension, training-set size, and target number. The polynomial growth is exhibited, and the cubic or exponential growth with measurement set size $M_{k}$ is not exhibited. Then the overall complexity of PMBM filter is not dominated by these additional modules.

As a result, the main computational bottleneck remains as data association in the update stage, and the cubic growth with association scale is retained.

#### 3.5.2. Data Association Complexity with and Without Gating

In the PMBM update, the computational complexity is dominated by data association. A *K*-best assignment strategy, Murty’s method [29], is used to generate near-optimal association hypotheses. Under the dense cost-matrix assumption, retaining $A_{k}$ global hypotheses and generating $K_{k}$ association solutions per hypothesis results in an overall association complexity $C_{ac}=O\left(A_{k}K_{k}{\left(N_{k}+M_{k}\right)}^{3}\right)$. Since $M_{k}$ typically increases approximately linearly with clutter density, this term becomes the computational bottleneck in dense-clutter scenarios.

When measurement gating is introduced, only measurements that pass the gate participate in data association. Suppose ${\tilde{M}}_{k}\leq M_{k}$ denote the effective number of measurements after gating. The complexity becomes $C_{gac}=O\left(A_{k}K_{k}{\left(N_{k}+{\tilde{M}}_{k}\right)}^{3}\right)$, the measurement set compression is defined as $\Delta M_{k}=M_{k}-{\tilde{M}}_{k}$ with $\Delta M_{k}\geq 0$, and the complexity reduction can be characterized:

(86) $$\Delta C_{ac}≜C_{ac}-C_{gac}=A_{k}K_{k}\left[{\left(N_{k}+M_{k}\right)}^{3}-{\left(N_{k}+{\tilde{M}}_{k}\right)}^{3}\right]$$

Substituting $\Delta M_{k}=M_{k}-{\tilde{M}}_{k}$ and expanding gives

(87) $$\Delta C_{assoc}=A_{k}K_{k}\left[3{\left(N_{k}+M_{k}\right)}^{2}\Delta M_{k}-3{\left(N_{k}+{\tilde{M}}_{k}\right)}^{2}{\left(\Delta M_{k}\right)}^{2}+{\left(\Delta M_{k}\right)}^{3}\right]$$

Note that $\Delta M_{k}\geq 0$ implies a reduction in association complexity, and the dominant term $3{\left(N_{k}+M_{k}\right)}^{2}\Delta M_{k}$ shows that a linear decrease in measurement set size produced by gating is amplified quadratically in the data-association cost.

The impact of the proposed adaptive gate and the conventional chi-square ellipsoidal gate on overall complexity is evaluated through the expected number of in-gate clutter measurements. Under a Poisson clutter model, the expected in-gate clutter count satisfies $E\left(M_{k}^{c}\right)=\lambda_{c}V_{g}$ where $\lambda_{c}$ is the clutter rate and $V_{g}$ is the gate volume. For the same designed gating probability, the proposed adaptive gate yields a smaller $V_{g}$ than the conventional chi-square ellipsoidal gate, thereby reducing in-gate clutter and the effective measurement set size for association.

## 4. Simulation Experiments

In this section, the performance of proposed filter is evaluated under identical target-motion scenario with different clutter conditions. Simulations are used to assess tracking performance when true measurements are generated by nonlinear measurement function unavailable to filter. Compared filters are tested under identical motion, detection, and clutter settings. All methods are implemented within GM-PMBM framework.

### 4.1. Simulation Settings

The sensor is assumed to operate in a two-dimensional Cartesian coordinate region $\left[-2000,2000\right]\times \left[0,2000\right]$ $m^{2}$, where ten distinct targets are present during each measurement interval. The sampling interval of ΔT=1 s is used, and 100 Monte Carlo trials are performed over 100 s. For each target, the motion state is modeled as ${\hat{x}}_{k}=\left[x_{k},{\dot{x}}_{k},y_{k},{\dot{y}}_{k},\omega_{turn}\right]$, where $\left(x_{k},y_{k}\right)$ denote the position in the turn rate with standard deviation $\sigma_{turn}=\pi /90{}^{\circ}$; the start and stop times are defined as $\left(t_{start},t_{stop}\right)$. Two motion modes are considered, constant velocity (CV) and coordinated turn (CT), and the corresponding state-transition matrix is defined as

(88) $$F_{\mathrm{CV}}=\left[\begin{array}{cccc} 1 & 1 & 0 & 0\\ 0 & 1 & 0 & 0\\ 0 & 0 & 1 & 1\\ 0 & 0 & 0 & 1 \end{array}\right]$$

(89) $$F_{\mathrm{CT}}=\left[\begin{array}{cccc} 1 & \sin\omega_{turn}/\omega_{turn} & 0 & \left(\cos\omega_{turn}-1\right)/\omega_{turn}\\ 0 & \cos\omega_{turn} & 0 & -\sin\omega_{turn}\\ 0 & 1-\cos\omega_{turn} & 1 & \sin\omega_{turn}/\omega_{turn}\\ 0 & \sin\omega_{turn} & 0 & \cos\omega_{turn} \end{array}\right]$$

For the CV and CT motion modes, the process-noise covariance matrices are set identically as

(90) $$Q_{\mathrm{CV}}=Q_{\mathrm{CT}}=\left[\begin{array}{cccc} 1/4 & 1/2 & 0 & 0\\ 1/2 & 1 & 0 & 0\\ 0 & 0 & 1/4 & 1/2\\ 0 & 0 & 1/2 & 1 \end{array}\right]\sigma_{p}^{2}$$

The measurements are generated by a nonlinear measurement function and a state-dependent measurement-noise covariance:

(91) $$h_{k}^{*}=\left[\begin{array}{c} \sqrt{{x_{k}}^{2}+{y_{k}}^{2}}+0.01\sin\left(0.005x_{k}\right)\\ \mathrm{atan}2(x_{k},y_{k}) \end{array}\right]$$

(92) $$R_{k}^{*}=\left[\begin{array}{cc} {\left(3+0.002c_{k}\right)}^{2} & 0\\ 0 & {\left(0.5^{{}^{\circ}}+0.0002c_{k}\right)}^{2} \end{array}\right]$$

(93) $$c_{k}=\sqrt{{x_{k}}^{2}+{y_{k}}^{2}}$$

The measurement function $h_{k}^{*}$ and the true covariance $R_{k}^{*}$ are only used to generate observations in simulation and are not directly available.

For surviving targets, the survival probability is set to $p_{s}=0.98$, and the detection probability is set to $p_{D}=0.98$. For newborn targets, the birth parameter set $\pi_{b}={\left\{\left(r_{b}^{l},p_{b}^{l}\right)\right\}}_{l=1}^{4}$ is

(94) $$\left\{\begin{array}{l} r_{b}^{\left(1\right)}=0.02,\;m_{b}^{\left(1\right)}={\left[250,0,1500,0\right]}^{T}\\ r_{b}^{\left(2\right)}=0.02,\;m_{b}^{\left(2\right)}={\left[-750,0,1100,0\right]}^{T}\\ r_{b}^{\left(3\right)}=0.03,\;m_{b}^{\left(1\right)}={\left[-500,0,250,0\right]}^{T}\\ r_{b}^{\left(4\right)}=0.03,\;m_{b}^{\left(1\right)}={\left[750,0,500,0\right]}^{T} \end{array}\right.$$

In the simulations, the optimal subpattern assignment (OSPA) [30] distance is used as the performance metric, where both cardinality and localization errors are accounted for and larger values indicate lower accuracy. The order parameter is set to p=2 and the cutoff parameter is set to c=100 m.

### 4.2. GP Regression Settings

The GP regression is implemented using squared-exponential kernel in Equation (30), and the hyperparameter is defined as $\Theta =\left\{\sigma_{x}^{2},I,\sigma_{v}^{2}\right\}$. Further, the regression-noise variance is set to $\sigma_{m}^{2}=\sigma_{v}^{2}$. The consistency with measurement-noise level used in simulations is maintained. The computational complexity is controlled by sliding-window training set with fixed length *L =* 50 at each time step:

(95) $$D=\left\{X,Z\right\}={\left\{\left(X_{i},Z_{i}\right)\right\}}_{i=1}^{L}$$

where $X_{i}\in R^{d_{x}}$ denotes a historical state sample. $Z_{i}\in R^{d_{z}}$ denotes the associated measurement sample. In GP implementation, $X_{i}$ is set to the predicted state mean of the associated Bernoulli component. is set to measurement assigned to that component at same time step. Samples are taken from previous *L* time steps only. Measurements not confirmed by data association and clutter samples are excluded from the GP training set. For two-dimensional measurement, two independent scalar GP models are trained for two measurement channels. Therefore, GP update is simplified, and the coupling parameters in output space are avoided.

When available sample number is smaller than $L_{\min}$, nominal surrogate model $\left(H_{GP,0},0,R_{GP,0}\right)$ is used. When available sample number reaches $L_{\min}$, GP model is activated. The updates are performed in sliding-window manner. Kernel hyperparameters $\Theta =\left\{\sigma_{x}^{2},I\right\}$ are estimated from Equation (35) at each time step. Maximum number of optimization iterations is set to 50. All GP-based methods in comparison use identical kernel form, training-window length, activation rule, and hyperparameter-estimation settings. Fair comparison is ensured. Consistency with complexity analysis in above is maintained. GP-related complexity is dominated by training-set size *L*, state dimension, and number of associated Bernoulli components.

### 4.3. Simulation Results

**Figure 2:**
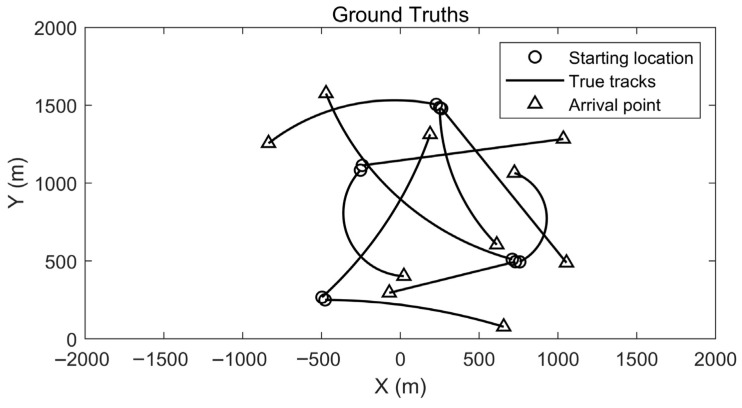
True trajectories of moving targets.

The simulation scenario contains ten moving targets that appear sequentially over time within the observation region, and the setting is designed to reflect common motion patterns and dynamic variations in road-traffic environments with practical relevance. The initial kinematic states, turn rates, and appearance/disappearance times of all targets are specified in Table 1. The target colors in the experiment are randomly assigned that are the same in related figures. When the turn rate $\omega_{trun}=0$, target motion is described by the CV model, and when $\omega_{trun}\neq 0$, the CT model is used with $\omega_{turn}=\pi /180{}^{\circ}$. The ground-truth trajectories are shown in Figure 2, where O and Δ mark the start and end points of each trajectory and the x- and y-axes correspond to the horizontal and vertical coordinates in the two-dimensional plane, the black curves represent the true target paths. Trajectory intersections may occur during motion, which increases the difficulty of data association, and in the presence of clutter the sensor-generated measurement set contains both true detections and clutter returns that are not directly separable at the observation level.

**Figure 3:**
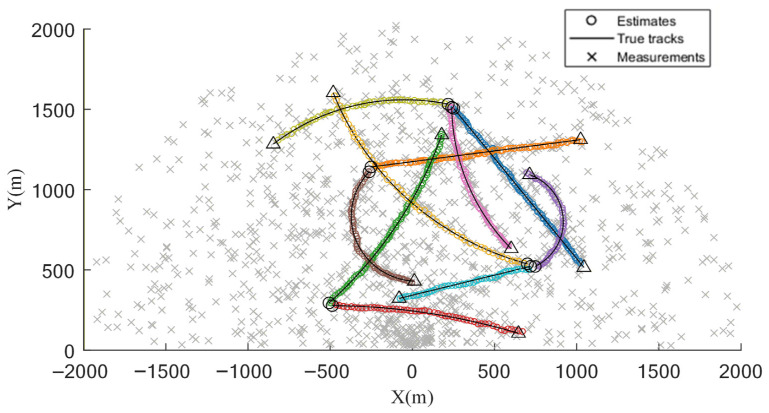
Target trajectories and available measurements.

The adaptive gating strategy is constructed by learning the predicted measurement distribution via GP regression, and the Bayesian measurement-origin test is used to probabilistically reweight measurements during the update step, which improves the suppression of clutter-induced interference. To examine robustness under heavy clutter, the clutter rate is set to λ=20 in the simulations. As illustrated in Figure 3, results over 100 Monte Carlo runs show that the estimated trajectories remain highly consistent with the ground truth across the surveillance region, indicating accurate state estimation and stable tracking in complex environments.

**Figure 4:**
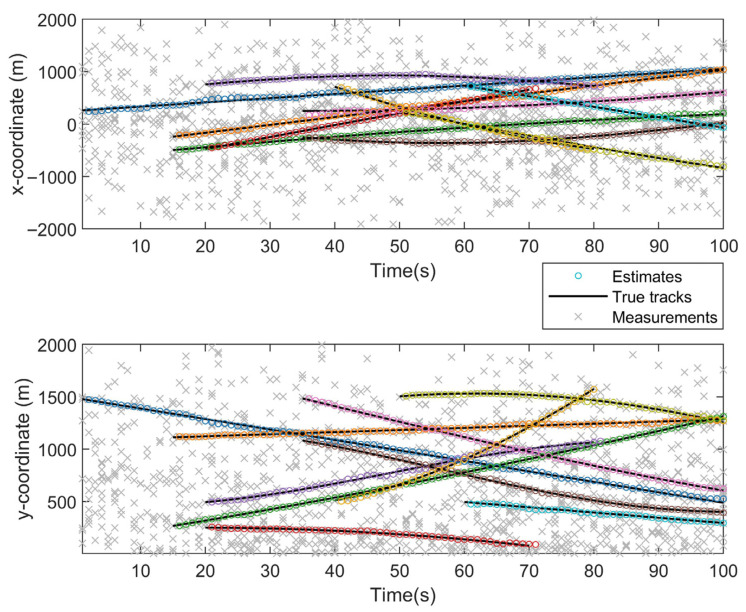
Target position estimates in x and y coordinates.

Figure 4 depicts the temporal evolution of the true trajectories and the estimated positions. With the sensor located at the origin, a denser clutter distribution is observed in its vicinity, and stronger interference is encountered in this area; increasing clutter density leads to larger local deviations between the estimated and true trajectories. Distinct targets exhibit different speeds and turn rates, and stronger maneuvering with higher motion speed is associated with increased filtering difficulty and slightly larger estimation errors. These characteristics are also reflected in Figure 2 and Figure 3, where the estimated trajectories remain stably distributed within a bounded neighborhood of the true trajectories without noticeable divergence or track loss. The behavior observed in Figure 3 and Figure 4 is primarily attributed to the adaptive gate, which effectively suppresses spurious measurements in dense-clutter scenarios. For the SVD-based and fading-factor-enhanced SCKF implementation, it provides numerical stability and robustness to model uncertainty during the update process.

**Figure 5:**
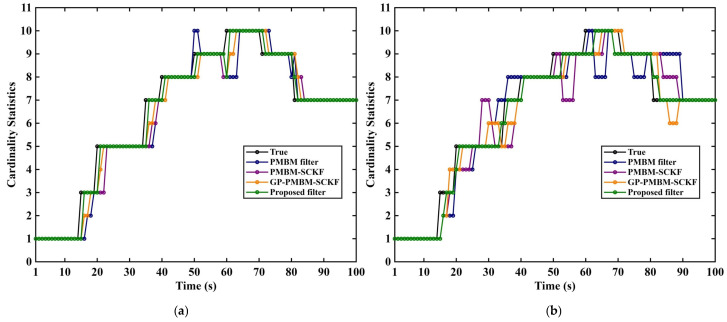
Target cardinality estimates under different clutter rates. (**a**) Noise $\left(\lambda =10\right)$. (**b**) Noise $\left(\lambda =20\right)$.

**Figure 6:**
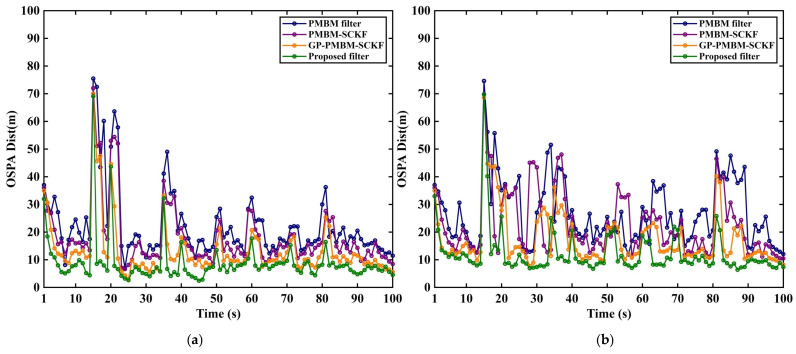
OSPA distance under different clutter rates. (**a**) Noise $\left(\lambda =10\right)$. (**b**) Noise $\left(\lambda =20\right)$.

**Figure 7:**
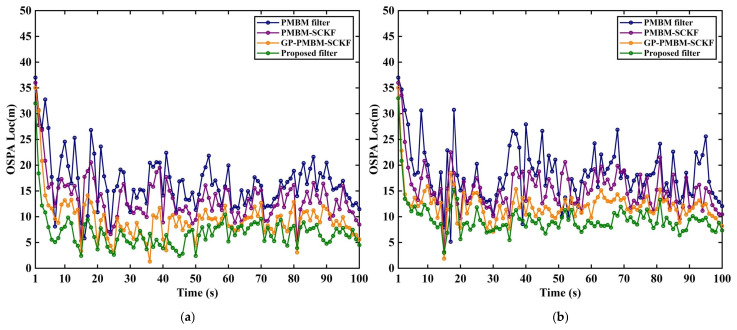
OSPA location distance under different clutter rates. (**a**) Noise $\left(\lambda =10\right)$. (**b**) Noise $\left(\lambda =20\right)$.

**Figure 8:**
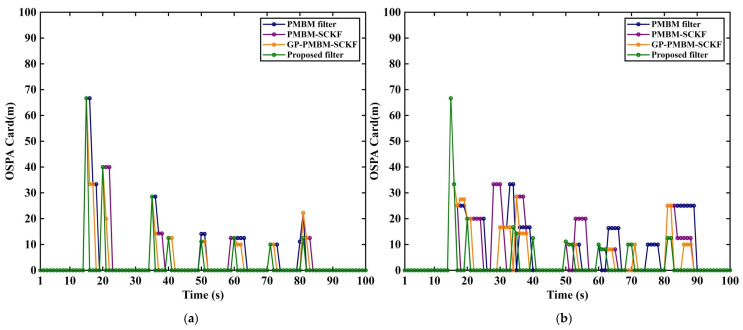
OSPA cardinality distance under different clutter rates. (**a**) Noise $\left(\lambda =10\right)$. (**b**) Noise $\left(\lambda =20\right)$.

Three representative filters, including GM-PMBM filter [31], GM-PMBM-SCKF [32], and GP-PMBM-SCKF, are evaluated alongside the proposed filter. The filter colors in the experiment are randomly assigned that are the same in related figures. Two average clutter rates are considered: λ=10 in Figure 5a, Figure 6a, Figure 7a and Figure 8a to represent a low-clutter setting, and λ=20 in Figure 5b, Figure 6b, Figure 7b and Figure 8b to represent a high-clutter setting.

Figure 5 shows cardinality estimation results of each filter under two clutter conditions. In Figure 5a, the cardinality estimates produced by all methods track true target number with small differences between curves. The proposed method stays closer to true cardinality. Smaller fluctuation magnitude can be observed. In Figure 5b, the clutter level is increased. More pronounced cardinality fluctuation and bias are observed for PMBM filter and PMBM-SCKF without GP-based measurement modeling. Overestimation and underestimation are amplified near target births, target deaths, and track intersections. Sensitivity to false alarms and association errors is increased under high clutter. The premature track termination is produced. With GP introduced, improved stability of cardinality estimation is obtained for GP-PMBM-SCKF. Further improvement is obtained when GP-driven predicted measurement distribution, adaptive ellipsoidal gating, and in-gate measurement-origin discrimination are included. Clutter-driven misassociation is suppressed effectively under high clutter. False births and erroneous terminations are reduced. Cardinality estimates become smoother and closer.

**Figure 9:**
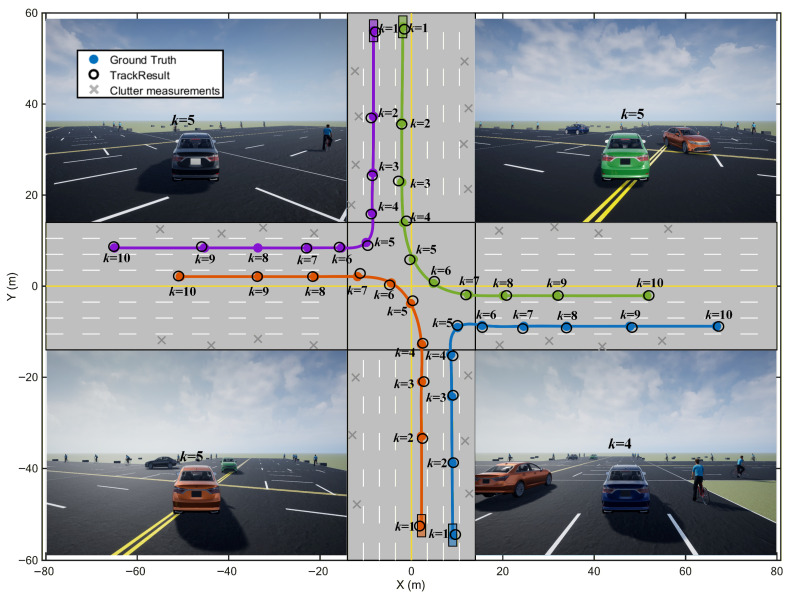
Tracking performance of proposed filter in an autonomous driving intersection scenario (unit: s).

The OSPA total distance and its localization and cardinality components are reported under different clutter rates (Figure 8 and Figure 9). Overall, pronounced peaks are observed around 15 s, 20 s, and 35 s in the OSPA curves, which is consistent with the time intervals where multiple targets enter the scene and where trajectory crossings occur, as indicated in Table 1. States of multi-target, step changes in the true target number occur at these times. The ambiguity in measurement-to-track association and newborn-target initialization is increased, which temporarily enlarges the total OSPA error. Additional fluctuations are observed around 70~80 s.

Figure 6 reports the OSPA total distance of each filter under two clutter conditions. In Figure 6a, the low-clutter level is considered. The total distances are close across methods. Similar average error levels are observed for the PMBM filter and PMBM-SCKF. Weak data-association disturbance is implied under low clutter, and overall impact from hypothesis generation and pruning is not pronounced. Smoother segments are observed for PMBM-SCKF. More stable handling of nonlinear state propagation is indicated. More accurate mean and covariance propagation is suggested. Lower OSPA levels are achieved by GP-PMBM-SCKF and the proposed method under low clutter. In Figure 6b, the clutter level is increased. Differences between methods become larger. Higher OSPA total distance is produced by the PMBM filter. Stronger fluctuation is observed. Error peaks are more frequent near target births, target deaths, and close target encounters. Some improvement is obtained by PMBM-SCKF relative to PMBM filter. Reduced association bias induced by measurement-model mismatch is indicated after predicted measurement distribution is constructed by GP. The performance under high clutter is achieved by proposed method when GP-driven adaptive ellipsoidal gating and in-gate measurement-origin discrimination are used. Stable error level is retained near 15 s, 20 s, and 35 s. Reduced clutter measurements entering association stage is implied by adaptive gating.

Figure 7 reports the OSPA localization distance of each filter under two clutter conditions. In Figure 7a, the low-clutter level is considered. Higher localization error is observed for PMBM filter and PMBM-SCKF. Stronger curve fluctuation is observed. Lower localization error is obtained for GP-PMBM-SCKF. Lowest OSPA localization distance is obtained for the proposed method. Lower-error region is maintained at most time steps. This behavior is attributed to improved square-root covariance construction and propagation in SVD-SCKF. Better numerical stability under nonlinear propagation is provided by SVD factorization relative to conventional implementation. More reliable predicted mean and covariance are obtained. Further, the adaptive fading factor improves responsiveness to model mismatch and maneuver variations. In Figure 7b, the higher clutter level is considered. Larger differences are observed in localization component. The highest OSPA localization distance is produced by PMBM filter, and the strongest fluctuation is observed. Larger peaks occur more often near target births, close target encounters, and pronounced maneuver changes. Some improvement is obtained by PMBM-SCKF relative to PMBM filter. Further reduction is obtained for GP-PMBM-SCKF. As a result, the lowest localization error level is retained by proposed filter under high clutter. Smoother curve is obtained. The improved numerical stability of square-root covariance construction and propagation is indicated for SVD-SCKF. The adaptive fading factor inflates predicted covariance under model mismatch or increased maneuvering. More accurate and stable localization results are maintained under high clutter.

In Figure 8a, the low-clutter condition is considered. The error peaks are concentrated near abrupt changes in target number. Obviously, the transient peaks are still observed for proposed method at some birth instants. Faster return to near-zero level is observed. Stronger suppression of cardinality disturbance is indicated. In Figure 8b, the high-clutter condition is considered. Larger differences are observed in OSPA cardinality Error. More nonzero peaks are produced by PMBM filter, PMBM-SCKF, and GP-PMBM-SCKF. Longer peak duration is observed in several intervals. Increased occurrence of false births, erroneous terminations, and delayed cardinality adjustment is indicated under high clutter. Then the proposed method still shows occasional large transient peaks at some birth instants. Moreover, fewer nonzero peaks are observed in subsequent intervals. Shorter duration is maintained. Note that the improvement is attributed mainly to GP-driven adaptive ellipsoidal gating. Fixed ellipsoidal gate is constructed directly from predicted measurement mean and covariance. The gate shape and scale remain unchanged. More in-gate clutter measurements are admitted near target births and close track encounters. The candidate association set expands rapidly. In-gate clutter measurements participating in PPP update can increase weights of false-birth hypotheses. Additional Bernoulli components can be created. During target disappearance, erroneous associations can delay decay of existing Bernoulli components. And the cardinality error can persist. In proposed filter, the predicted measurement mean and predicted measurement covariance are obtained from GP. Adaptive ellipsoidal gate is constructed from these quantities. Better match to current predicted measurement distribution is provided. Invalid measurements entering candidate association set are reduced. In-gate clutter reduction decreases birth hypotheses triggered by clutter in PPP update. During target disappearance, interference of erroneous associations on existence probability is weakened. As a result, the recovery of cardinality estimation is obtained.

Table 2 reports the average runtime of the four filters over 100 Monte Carlo runs under two clutter levels, $\lambda_{c}=10$ and $\lambda_{c}=20$. Increased clutter rate produces larger measurement sets. Higher data-association computational complexity is produced in the update stage. Runtime is increased for all methods. Shortest runtime is obtained by GM-PMBM-filter under both clutter levels. Increased runtime is observed for GM-PMBM-SCKF after SCKF is introduced. Higher computational complexity is indicated for nonlinear state propagation. Under $\lambda_{c}=10$, runtime of GP-GM-PMBM-SCKF is slightly lower than GM-PMBM-SCKF. Difference is small. Under $\lambda_{c}=20$, runtime of GP-GM-PMBM-SCKF is increased further. Largest runtime among four methods is obtained. Increased computational burden from GP-related processing is indicated under high clutter.

Lower runtime is obtained by proposed filter under both clutter levels relative to GM-PMBM-SCKF and GP-GM-PMBM-SCKF. Under $\lambda_{c}=20$ average runtime of proposed filter is 7.31 s. Shorter runtime is obtained than 8.26 s of GM-PMBM-SCKF and 8.42 s of GP-GM-PMBM-SCKF. Reduced number of invalid clutter measurements entering association stage is implied by GP-based adaptive ellipsoidal gating and in-gate measurement-origin discrimination. Reduced data-association computational complexity is obtained under high clutter.

To evaluate the effects of different modules, four variants are constructed. M0 denotes the standard GM-PMBM-SCKF. M1 denotes GM-PMBM-SCKF equipped with the SVD-based prediction module with an adaptive fading factor. M2 denotes GM-PMBM-SCKF equipped with the GP learning of measurement function module. M3 denotes the GP-based measurement processing variant, which extends M2 by further introducing the GP-driven adaptive ellipsoidal gating and the in-gate Bayesian measurement-origin discrimination, but does not include the SVD-based prediction module. The full proposed filter combines the modules used in M1 and M3.

Table 3 and Table 4 report results under different detection probabilities and clutter intensities. Metrics include generalized optimal subpattern assignment (GOSPA), localization error (LE), missed error (ME), and false error (FE). Note that the performance degradation for all four variants when detection probability is reduced or clutter intensity is increased. Increased impact from missed detections, false alarms, and association errors is indicated under low detection and heavy clutter. Lower LE is obtained by M1 than M0 under all parameter settings. Improved numerical stability in prediction stage is indicated for SVD-based square-root propagation. Improved responsiveness under model mismatch is indicated for adaptive fading factor. Localization error reduction is emphasized. Lower GOSPA is obtained by M2 than M1. Improvement is concentrated in ME and FE. Reduced association bias induced by measurement-model mismatch is indicated after GP-based measurement learning is introduced. Lower GOSPA is obtained by M3 than M2 under all parameter settings. Further reduction is concentrated in FE. ME is reduced to some extent. Reduced invalid measurements entering association set is indicated for GP-based adaptive ellipsoidal gating. Misassociations and false births are suppressed. The lowest GOSPA is maintained by M3 under all test conditions. More stable and more accurate multi-target tracking is obtained under low detection probability and dense clutter when GP-based measurement learning and GP-driven adaptive gating are combined within GM-PMBM-SCKF framework.

### 4.4. Tracking Simulation Results Under Highly Maneuvering Scenarios

To qualitatively describe the detection performance of proposed filter for actual scenario, it is integrated into the Automated Driving Toolbox as a custom multi-object tracker, where recursive estimation is performed at each simulation step using simulated measurements and target trajectories are generated for tracking validation in simulation scenarios. Figure 9 shows the tracking results of proposed filter in the traffic scenario. The vehicle colors in the experiment are randomly assigned. Four vehicles enter turning segment simultaneously at *k* = 5 s. Small spatial separations are present near intersection center at same time step. Higher requirements are imposed on track discrimination and turn response. Estimated trajectories are consistent with ground truth at turn entry, mid-turn, and post-turn segments. The trajectory continuity and target resolvability are maintained during simultaneous maneuvers. Numerical stability of square-root covariance construction and propagation is improved by SVD-SCKF, where the predicted covariance is inflated by adaptive fading factor under model mismatch or increased maneuvering, and the state bias is corrected more promptly at turn onset. Consistency between measurement modeling in the update stage and sensing process is improved by GP-based measurement function learning. Obviously, under the clutter environments, the vehicles tracking results are quite satisfactory.

## 5. Discussion

The proposed method is developed within the PMBM filtering framework, where an SVD-based square-root implementation is adopted in the SCKF to replace the Cholesky decomposition and the GP is incorporated into measurement modeling and the update stage to mitigate the impact of measurement-model mismatch. Experimental results indicate that as system nonlinearity increases, the detection probability decreases, and the clutter density rises, performance degradation is more gradual, and error curves exhibit smaller fluctuations. In addition, the extensibility is discussed from two aspects based on existing literature, including the measurement model and computational complexity.

At the measurement-model level, the GP in existing frameworks is typically used to learn the nonlinear mapping between the state and the measurement and to characterize how measurement uncertainty varies with the state. For extended targets, the measurement model is no longer based on a single-point return assumption. The spatial distribution of measurement clusters induced by target extent and shape is required to be represented. The extension can therefore be formulated from the measurement-model perspective by augmenting each Bernoulli component in the PMBM posterior with shape parameters in addition to the kinematic state, where star-convex shapes, multidimensional splines, random triangles, and other complex geometric representations may be included. By using the GP to model in a data-driven manner the relationship between the extent-augmented target state and the statistical characteristics of the measurement cluster, the resulting measurement likelihood reflects the effects of both target position and extent on the measurement distribution.

With respect to the computational complexity level, standard GP regression requires repeated kernel-matrix operations whose size grows with the number of accumulated samples. The learning stage is prone to becoming a computational bottleneck when the training set keeps expanding in online tracking. Sparse approximations such as sparse variational GP can be adopted to compress informative content into a fixed number of inducing points or a low-rank structure. The hyperparameters and predictive distributions can be updated via mini-batch stochastic optimization. As a result, the per-step cost is mainly scaled by the number of inducing points rather than by the number of historical samples; tighter approximations, including orthogonal variational formulations, can further improve approximation quality and inference stability under the same computational budget. For continuously arriving data streams, streaming sparse GP recursions are used to assimilate new samples while retaining previously learned information, and the sliding-window scheme or forgetting strategy can be combined to emphasize recent operating conditions, keeping the long-run computational cost within an acceptable range.

## 6. Conclusions

A novel multi-target tracking method based on the proposed filter is presented for nonlinear measurement environments with model mismatch and dense clutter. In the prediction stage, the SVD-based SCKF propagation scheme with an adaptively determined fading factor is applied to inflate the covariance, improving numerical stability and tracking responsiveness under maneuvering conditions. In the update stage, the GP is trained to learn the measurement mapping and its state-dependent uncertainty, providing data-driven support for constructing the measurement likelihood, computing association weights, and performing Bayesian updates. The adaptive gate is constructed from the GP-driven predictive measurement distribution and an in-gate binary Bayesian measurement-origin test is used so clutter-like measurements are assigned smaller weights in association and update. Simulation results indicate that, in challenging multi-target scenarios, improved estimation accuracy and stronger numerical stability are achieved compared with existing methods, particularly for highly maneuvering targets.

## Figures and Tables

**Table 1:** States of multi-target.

Target Label	Initial State (m, m/s)	Angular Velocity (rad/s)	Starting and Ending Time (s)
1	[253, 10, 1488, −10]^T^	0	(1, 101)
2	[−255, 15, 1111, 2]^T^	0	(15, 101)
3	[507, 11, 257, 10]^T^	*ω*_turn_/6	(15, 101)
4	[500, 23, 250, 0]^T^	*ω*_turn_/6	(20, 70)
5	[746, 11, 489, 5]^T^	9*ω*_turn_/10	(20, 80)
6	[−243, 10, 1094, −10]^T^	*ω*_turn_/1	(35, 101)
7	[250, 0, 1500, −15]^T^	*ω*_turn_/3	(35, 101)
8	[750, −40, 500, 10]^T^	−3*ω*_turn_/2	(40, 80)
9	[250, −22, 1500, −15]^T^	*ω*_turn_/2	(50, 101)
10	[750, −20, 500, −5]^T^	0	(60, 101)

**Table 2:** Average computational times (s).

$\lambda_{c}$	GM-PMBM Filter	GM-PMBM-SCKF	GP-GM-PMBM-SCKF	Proposed Filter
10	4.71	5.45	5.31	5.22
20	7.17	8.26	8.42	7.31

**Table 3:** Under different parameter settings, root mean square GOSPA (m) and LE, ME, FE of M0-PMBM and M1-PMBM.

($P_{D},\lambda_{c}$)	M0-PMBM				M1-PMBM			
	GOSPA	LE	ME	FE	GOSPA	LE	ME	FE
(0.98, 10)	2.7493	1.7756	1.9282	0.8281	2.6971	1.7242	1.9122	0.8040
(0.98, 15)	2.7908	1.7963	1.9581	0.8532	2.7587	1.7391	1.9671	0.8463
(0.98, 20)	2.8238	1.8011	1.9691	0.9234	2.7898	1.7562	1.9732	0.8971
(0.90, 10)	2.7940	1.7967	1.9671	0.8417	2.7778	1.7762	1.9632	0.8406
(0.90, 20)	2.8732	1.8011	2.0120	0.9817	2.8422	1.7983	1.9875	0.9456
(0.68, 10)	3.8715	2.2153	2.9391	1.2011	3.8219	2.1214	2.8431	1.1261
(0.68, 20)	4.2987	2.2431	3.4104	1.3475	4.1371	2.1435	3.3015	1.2731

**Table 4:** Under different parameter settings, root mean square GOSPA (m) and LE, ME, FE of M2-PMBM and M3-PMBM.

($P_{D},\lambda_{c}$)	M2-PMBM				M3-PMBM			
	GOSPA	LE	ME	FE	GOSPA	LE	ME	FE
(0.98, 10)	2.6155	1.7613	1.7932	0.7234	2.5397	1.7525	1.7241	0.6374
(0.98, 15)	2.6701	1.7883	1.8163	0.7952	2.5756	1.7762	1.7431	0.6633
(0.98, 20)	2.7323	1.8156	1.8576	0.8477	2.6483	1.8021	1.7972	0.7322
(0.90, 10)	2.7389	1.8457	1.8542	0.8105	2.6661	1.8331	1.8011	0.7102
(0.90, 20)	2.8045	1.8721	1.8932	0.8812	2.7417	1.8930	1.8261	0.7738
(0.68, 10)	3.7322	2.2362	2.7806	1.0941	3.5658	2.2147	2.6231	0.9642
(0.68, 20)	4.0416	2.2561	3.1435	1.1678	3.9652	2.3011	3.0452	1.0744

## Data Availability

No new data were created or analyzed in this study. Data sharing is not applicable to this article.

## Abbreviations

Y	target state vector
z	measurement vector
f(·)	state-transition function
h(·)	measurement function
w	process noise
v	measurement noise
Q	process-noise covariance
R	measurement-noise covariance
X	multi-target state set
Z	measurement set
$f^{p}(\cdot )$	Poisson RFS density
$f^{MBM}(\cdot )$	multi-Bernoulli mixture (MBM) RFS density
$\kappa \left(z\right)$	clutter intensity function
r	Bernoulli existence probability
p(·)	single-target state density
$w^{j}$	weight of global hypothesis j
$I^{j}$	index set of Bernoulli components in hypothesis j
$X_{i}$	i-th cubature point
$X_{i}^{*}$	propagated cubature point through the state-transition function
$P^{*}$	covariance matrix in expectation form
P	state covariance matrix
$\lambda_{i}$	fading factor
K	Kalman gain
K	GP kernel matrix
θ	GP hyperparameter set
$\beta^{b}(\cdot )$	GM birth intensity for newborn targets
$J^{b}$	number of Gaussian components in the birth intensity
$w^{b,m}$	weight of the m-th Gaussian component in the birth intensity
${\bar{x}}^{b,m}$	mean of the m-th Gaussian component in the birth intensity
$P^{b,m}$	covariance matrix of the m-th Gaussian component in the birth intensity
G	gating region
γ	gating threshold
$\lambda_{c}$	Poisson clutter rate
$\pi_{k}(z)$	normalized predictive density
CPHD	Cardinalized Probability Hypothesis Density
GOSPA	Generalized Optimal Subpattern Assignment
GLMB	Generalized Labeled Multi-Bernoulli
GP	Gaussian Process
KLD	Kullback–Leibler Divergence
LMB	Labeled Multi-Bernoulli
OSPA	Optimal Subpattern Assignment
SCKF	Square-Root Cubature Kalman Filter
SMC	Sequential Monte Carlo
SVD	Singular Value Decomposition

## Appendix A. Robustness of SVD Implementation

Since the state covariance matrix P is symmetric positive semidefinite, an orthogonal matrix U exists such that $A^{T}PA\geq 0$ holds for any nonzero vector A, and the SVD in can be written as

(A1) $$P=U\Sigma U^{T}$$

where Σ is the diagonal matrix of singular values defined as

(A2) $$\begin{array}{cc} \Sigma =\mathrm{diag}\left(\sigma_{1},\sigma_{2},\cdots ,\sigma_{n}\right), & \sigma_{1}\geq \sigma_{2}\geq \cdots \geq \sigma_{r}>\sigma_{r+1}=\cdots =\sigma_{n} \end{array}=0$$

The covariance after considering ΔP perturbation is

(A3) $$\tilde{P}=P+\Delta P$$

According to Weyl’s theorem, the singular-value perturbation satisfies

(A4) $$\left|{\tilde{\sigma }}_{i}-\sigma_{i}\right|\leq {\left\|\Delta P\right\|}_{2}\leq {\left\|\Delta P\right\|}_{F}\leq \varepsilon$$

where ε is the perturbation error bound. The SVD of $\tilde{P}$ in (A3) is expressed as

(A5) $$\tilde{P}=\tilde{U}\tilde{\Sigma }{\tilde{U}}^{T}$$

where $\tilde{\Sigma }$ denotes the singular-value matrix of $\tilde{P}$, which is defined by the singular values ${\tilde{\sigma }}_{n}$ as

(A6) $$\tilde{\Sigma }=\mathrm{diag}\left({\tilde{\sigma }}_{1},{\tilde{\sigma }}_{2},\ldots ,{\tilde{\sigma }}_{n}\right)$$

In the SCKF, the covariance square-root is generated as $S=U\Sigma^{1/2}$. After perturbation, it becomes, and the $\tilde{S}=\tilde{U}{\tilde{\Sigma }}^{1/2}$ corresponding deviation is

(A7) $$\Delta S=\tilde{S}-S=\tilde{U}{\tilde{\Sigma }}^{1/2}-U\Sigma^{1/2}=\left(\tilde{U}-U\right)\Sigma^{1/2}+\tilde{U}\left({\tilde{\Sigma }}^{1/2}-\Sigma^{1/2}\right)$$

For the first term in (A7), the subspace perturbation term is obtained as

(A8) $${\left\|\left(\tilde{U}-U\right)\Sigma^{1/2}\right\|}_{F}={\left\|\left(\tilde{U}-U\right)\right\|}_{F}\|\Sigma^{1/2}{\|}_{2}\leq \frac{C\varepsilon }{\Delta \sigma }\sigma_{\max}^{1/2}$$

To ensure the stability of the eigensubspace, a spectral gap $\Delta \sigma =\underset{i\leq r,j\geq r+1}{\min}\left|\sigma_{i}-\sigma_{j}\right|>0$ is introduced via the Davis–Kahan theorem with a constant C>0; the square-root function is Lipschitz continuous on $\left[\sigma_{\min},\infty \right]$ and satisfies $\left|{\tilde{\sigma }}_{i}^{1/2}-\sigma_{i}^{1/2}\right|\leq \frac{1}{2\sigma_{\min}^{1/2}}\left|{\tilde{\sigma }}_{i}-\sigma_{i}\right|$. Then, the singular-value perturbation term in the second part of (33) is bounded by

(A9) $${\left\|\tilde{U}\left({\tilde{\Sigma }}^{1/2}-\Sigma^{1/2}\right)\right\|}_{F}={\left\|{\tilde{\Sigma }}^{1/2}-\Sigma^{1/2}\right\|}_{F}={\left(\sum_{i=1}^{n}{\left({\tilde{\sigma }}_{i}^{1/2}-\sigma_{i}^{1/2}\right)}^{2}\right)}^{1/2}\leq {\left(\sum_{i=1}^{n}{\left(\frac{\varepsilon }{2\sigma^{1/2}}\right)}^{2}\right)}^{1/2}=\frac{n^{1/2}\varepsilon }{2\sigma_{\min}^{1/2}}$$

To avoid ill-conditioning, suppose $\sigma_{\min}=\min\left\{\sigma_{i}>0\right\}$.

Combining (A8) and (A9) with (A6) yields

(A10) $${\left\|\Delta S\right\|}_{F}={\left\|\left(\tilde{U}-U\right)\Sigma^{1/2}\right\|}_{F}+{\left\|{\tilde{\Sigma }}^{1/2}-\Sigma^{1/2}\right\|}_{F}\leq \frac{C\varepsilon }{\Delta \sigma }\sigma_{\max}^{1/2}+\frac{n^{1/2}\varepsilon }{2\sigma_{\min}^{1/2}}$$

In the error propagation analysis of state estimation, when the cubature-point perturbation $\Delta X_{i}$ is taken into account, the formula for generating cubatures points is as follows:

(A11) $$\left\{\begin{array}{l} X_{i}=S\xi_{i}+\bar{x}\\ {\tilde{X}}_{i}=\tilde{S}\xi_{i}+\tilde{\bar{x}} \end{array}\right.$$

For a single point, the perturbation error is bounded by

(A12) $$\left\|\Delta X_{i}\right\|=\left\|\tilde{S}\xi_{i}-S\xi_{i}\right\|\leq {\left\|\tilde{S}-S\right\|}_{F}\left\|\xi_{i}\right\|\leq {\left\|\Delta S\right\|}_{F}\left\|\xi_{i}\right\|$$

For the nonlinear propagation perturbation, the state-transition function is assumed to satisfy a Lipschitz condition:

(A13) $$\left\|f\left({\tilde{X}}_{i}\right)-f\left(X_{i}\right)\right\|\leq L_{\phi }\left\|\Delta X_{i}\right\|\leq L_{\phi }{\left\|\Delta S\right\|}_{F}\left\|\xi_{i}\right\|$$

where $L_{\phi }$ is the Lipschitz constant.

Consequently, the prior state-estimation error admits the bound:

(A14) $$\left\|\Delta \bar{x}\right\|=\left\|\tilde{\bar{x}}-\bar{x}\right\|\leq \frac{1}{2n}\sum_{i=1}^{2n}L_{\phi }{\left\|\Delta S\right\|}_{F}\left\|\xi_{i}\right\|=L_{\phi }{\left\|\Delta S\right\|}_{F}\left\|\xi_{i}\right\|$$

Substituting (A8) and (A9) into (A14) gives the final bound:

(A15) $${\left\|\Delta S\right\|}_{F}={\left\|\left(\tilde{U}-U\right)\Sigma^{1/2}\right\|}_{F}+{\left\|{\tilde{\Sigma }}^{1/2}-\Sigma^{1/2}\right\|}_{F}\leq \frac{C\varepsilon }{\Delta \sigma }\sigma_{\max}^{1/2}+\frac{n^{1/2}\varepsilon }{2\sigma_{\min}^{1/2}}$$

Note that as ε→0 the state-estimation error upper bound converges to zero and is governed by the spectral properties of the system through $\sigma_{\min}$, Δσ and $L_{\phi }$ which provides a theoretical basis for replacing the Cholesky decomposition with SVD in SCKF under ill-conditioned scenarios.

## Appendix B. Pseudocode

This pseudocode presents SVD-SCKF-based prediction in GM-PMBM filter. Recursions are given for Gaussian components in PPP part and Bernoulli part. SVD square-root factorization is applied to covariance. Cubature points are generated. Predicted weights, predicted means, and predicted covariances are obtained for each component.

**Algorithm A1:** Prediction based on SVD-SCKF in GM-PMBM filter
**Input:** Birth PPP intensity $\beta_{k}^{b}\left(x\right)$; prior PPP intensity $\mu_{k-1}\left(x\right)$; Bernoulli components $\left\{w_{k-1}^{j,l},r_{k-1}^{j,l},p_{k-1}^{j,l}\left(x\right)\right\}$ survival probability $p_{S,k-1}$; process-noise covariance $Q_{k-1}$. **//PPP prediction (each GM component):** **for** $m=1,\ldots ,J_{k-1}^{\mu }$ **do** $\omega_{k|k-1}^{\mu ,m}=p_{S,k-1}\omega_{k-1}^{\mu ,m}$ Compute SVD square-root $\sqrt{P_{k-1}^{\mu ,m}}$ via (6)–(7), then form cubature points via (48) Propagate cubature points and compute $\left({\bar{x}}_{k|k-1}^{\mu ,m},P_{k|k-1}^{\mu ,m}\right)$ via (49)–(51). Compute $\mu_{k|k-1}\left(x\right)$ according to (46) **end for** **//Bernoulli prediction (each component):** **for** all Bernoulli components $\left(j,l\right)$ **do** Compute the predicted existence probability $r_{k|k-1}^{j,l}$ according to (53). Compute the predicted Bernoulli component weight $w_{k|k-1}^{j,l}$ according to (54). Compute SVD square-root $\sqrt{P_{k-1}^{j,l}}$ via (6) and (7), then form cubature points via (57). Propagate cubature points and compute $\left({\bar{x}}_{k|k-1}^{j,l},P_{k|k-1}^{j,l}\right)$ via (55) and (56). Compute $p_{k|k-1}^{j,l}\left(x\right)$ according to (52). **end for** **Output:** Predicted PPP intensity $\mu_{k|k-1}\left(x\right)$ and Bernoulli component $\left\{w_{k|k-1}^{j,l},r_{k|k-1}^{j,l},p_{k|k-1}^{j,l}\left(x\right)\right\}$.

## Appendix C. Pseudocode

This pseudocode presents the GP-aided cubature update in the GM-PMBM filter with sliding-window training set. GP-based predicted measurement computation and moment matching are performed in the update stage for PPP components and Bernoulli components. Historical state–measurement samples are used to update GP training set. Kernel matrix is precomputed. GP posterior is evaluated at each cubature point. Predicted measurement mean and covariance are obtained. Measurement-predicted mean, measurement covariance, state–measurement cross-covariance, and Kalman gain required for update are produced.

**Algorithm A2:** GP-aided cubature update for GM-PMBM filter with sliding-window training set
**Input:** Predicted PMBM parameters at *k*: PPP GM ${\left\{w_{k|k-1}^{m},{\bar{x}}_{k|k-1}^{\mu ,m},P_{k|k-1}^{\mu ,m}\right\}}_{m=1}^{J_{k|k-1}^{\mu }}$; Bernoulli set $\left\{w_{k|k-1}^{j,l},r_{k|k-1}^{j,l},{\bar{x}}_{k|k-1}^{j,l},P_{k|k-1}^{j,l}\right\}$; measurement set $Z_{k}$; clutter intensity κ(·); cubature directions ${\left\{\alpha_{i}\right\}}_{i=1}^{2n}$; window length *L*; previous GP training set $D_{k-1}=\{X_{k-1},Z_{k-1}\}$. **//Sliding-window update of GP training set** Append new training pairs into $D_{k-1}$ to form; discard the oldest pairs if $|D_{k}|\;>L$. //**Pre-compute GP matrices** Compute $K_{k}=K(X_{k},X_{k})$ and $W_{k}={\left(K_{k}+\sigma_{v}^{2}I\right)}^{-1}Z_{k}$. //**GP prediction at cubature points and moment matching** **for** PPP GM component $m=1,\cdots ,J_{k|k-1}^{\mu }$ **do** **points** $X_{i,k}^{p,m}={\bar{x}}_{k|k-1}^{\mu ,m}+\sqrt{P_{k|k-1}^{\mu ,m}}\alpha_{i},\;i=1,\cdots ,2n$**.** For each $X_{i,k}^{p,m}$, compute $K_{*,i}^{p,m}=K\left(X_{k},X_{i,k}^{p,m}\right)$ and $K_{**,i}^{p,m}=K\left(X_{i,k}^{p,m},X_{i,k}^{p,m}\right)$, then obtain the GP posterior prediction (cf. (59) and (60)): $z_{i,k}^{p,m}=\zeta (X_{i,k}^{p,m})={\left(K_{*,i}^{p,m}\right)}^{T}W_{k}$ and $R_{i,k}^{p,m}=\vartheta (X_{i,k}^{p,m})=K_{**,i}^{p,m}-{\left(K_{*,i}^{p,m}\right)}^{T}{\left(K_{k}+\sigma_{v}^{2}I\right)}^{-1}K_{*,i}^{p,m}$ Moment matching yields ${\bar{x}}_{k}^{m}=\frac{1}{2n}\sum_{i=1}^{2n}z_{i,k}^{p,m}$, $S_{k}^{m}=\frac{1}{2n}\sum_{i=1}^{2n}(z_{i,k}^{p,m}-{\bar{z}}_{k}^{m}){\left(z_{i,k}^{p,m}-{\bar{z}}_{k}^{m}\right)}^{T}+R_{i,k}^{p,m}$, $P_{xz,k}^{m}=\frac{1}{2n}\sum_{i=1}^{2n}(X_{i,k}^{p,m}-{\bar{x}}_{k|k-1}^{\mu ,m})-{\left(z_{i,k}^{p,m}-{\bar{z}}_{k}^{m}\right)}^{T}$ and $K_{k}^{m}=P_{xz,k}^{m}{\left(S_{k}^{m}\right)}^{-1}$. **end for** **for** Bernoulli component (*j*,*l*) **do**: Generate cubature points $X_{i,k}^{j,l}={\bar{x}}_{k|k-1}^{j,l}+\sqrt{P_{k|k-1}^{j,l}}\alpha_{i},\;i=1,\ldots ,2n$; Compute $K_{*,i}^{j,l}=K(X_{k},X_{i,k}^{j,l})$, $K_{**,i}^{j,l}=K\left(X_{i,k}^{j,l},X_{i,k}^{j,l}\right)$, $z_{i,k}^{j,l}=\zeta (X_{i,k}^{j,l})={\left(K_{*,i}^{j,l}\right)}^{T}W_{k}$, $R_{i,k}^{j,l}=\vartheta (X_{i,k}^{j,l})=K_{**,i}^{j,l}-{\left(K_{*,i}^{j,l}\right)}^{T}{\left(K_{k}+\sigma_{v}^{2}I\right)}^{-1}K_{*,i}^{j,l}$, and obtain ${\bar{x}}_{k}^{j,l}$, $S_{k}^{j,l}$, $P_{xz,k}^{j,l}$, $K_{k}^{j,l}$ by the same moment-matching formulas. **end for** **Output**: Updated PMBM posterior at *k*.
